# Polygenic Risk Score in African populations: progress and challenges

**DOI:** 10.12688/f1000research.76218.2

**Published:** 2023-04-11

**Authors:** Yagoub Adam, Suraju Sadeeq, Judit Kumuthini, Olabode Ajayi, Gordon Wells, Rotimi Solomon, Olubanke Ogunlana, Emmanuel Adetiba, Emeka Iweala, Benedikt Brors, Ezekiel Adebiyi

**Affiliations:** 1 Covenant University Bioinformatics Research (CUBRe), Covenant University, Ota, Ogun State, 112212, Nigeria; 2 Covenant Applied Informatics and Communication Africa Centre of Excellence (CApIC-ACE), Covenant University, Ota, Ogun State, 112212, Nigeria; 3 Dept Computer & Information Sciences, Covenant University, Ota, Ogun State, 112212, Nigeria; 4 South African National Bioinformatics Institute, Life Sciences Building, University of Western Cape, Cape Town, South Africa; 5 Centre for Proteomic and Genomic Research, Cape Town, Western Cape, South Africa; 6 Dept of Biochemistry, Covenant University, Ota, Ogun State, 112212, Nigeria; 7 Dept of Electrical & Information Engineering (EIE), Covenant University, Ota, Ogun State, 112212, Nigeria; 8 HRA, Institute for Systems Science, Durban University of Technology, Durban, South Africa; 9 Applied Bioinformatics Division, German Cancer Research Center (DKFZ), Heidelberg, 69120, Germany; 10 German Cancer Consortium (DKTK), Heidelberg, Germany

**Keywords:** Prediction medicine, GWAS, post-GWAS, PRS analysis, Africa population

## Abstract

Polygenic Risk Score (PRS) analysis is a method that predicts the genetic risk of an individual towards targeted traits. Even when there are no significant markers, it gives evidence of a genetic effect beyond the results of Genome-Wide Association Studies (GWAS). Moreover, it selects  single nucleotide polymorphisms (SNPs) that  contribute to the disease with low effect size  making it more precise at individual level risk prediction. PRS  analysis addresses the shortfall of GWAS by taking into account the SNPs/alleles with  low effect size but play an indispensable role to the observed phenotypic/trait variance.  PRS analysis has  applications that investigate the genetic basis of several traits, which includes rare diseases. However, the accuracy of PRS analysis depends on the genomic data of the underlying population. For instance, several studies  show   that obtaining higher prediction power of PRS analysis is challenging for non-Europeans. In this manuscript, we review the conventional PRS methods and their application to sub-Saharan African communities. We conclude that  lack of sufficient GWAS data and tools is  the limiting factor of applying PRS analysis to sub-Saharan populations.   We recommend developing Africa-specific PRS methods and tools for estimating and analyzing  African population data   for clinical  evaluation of PRSs of interest and predicting  rare diseases.

## Introduction

Genome-Wide Association Studies (GWAS) can be used successfully to identify associations between hundreds of genomic variations with complex genetic traits. ^1^ In general, GWAS report single nucleotides polymorphisms (SNPs) as statistically significant genomic variations associated with the trait of interest when their *p*-values are smaller than a cutoff value of 5e‐09 in the African population. ^2^ This cutoff value statistically depends on the number of SNPs analyzed. ^2^ The statistically significant SNPs reported by GWAS are used to understand the biomolecular mechanisms of many phenotypic traits including various human diseases. Due to the statistical threshold, GWAS might fail to detect SNPs that are associated with low or moderate risks. ^3^^,^^4^ The limitation of filtering variants associated with low disease risk increases the GWAS false-negative rate. Also, conventional GWAS can not be used to integrate the polygenic nature of many complex traits. ^5^ Therefore, several post-GWAS approaches have been introduced to overcome the above mentioned pitfalls. ^6^^,^^7^ Due to privacy issues, such as access to the individual level of GWAS data sets, most post-GWAS approaches require only GWAS summary statistics. Some public resources for GWAS summary statistics include: the GWAS Catalog, ^8^ GWAS Central, ^9^ and the dbGaP database. ^10^^,^^11^ A distinct approach of performing a post-GWAS analysis is known as Polygenic Risk Score (PRS) analysis. The PRS methods map genotype data from a GWAS summary into a single variable used to estimate an individual-level risk score for the phenotypic trait. PRS analysis is used to predict an individual heritability by incorporating all selected SNPs, ^12^ i.e., the proportion of trait variance (phenotype) that is associated with genetic variants (genotype). ^13^^,^^14^ However, it is important to consider that not all existing genomics technologies have the capabilities to capture the informative variants among trans-ethnic populations. Nevertheless, obtaining a precise PRS value from case-control studies can be used in personalized medicine. Challenges still exist when translating PRS values from clinical validity to clinical utility. ^15^ To successfully perform conventional PRS analysis, two distinct GWAS summaries are required. The first data set (training sample) is used to select the SNPs for PRS analysis and the second data set (from the discovery sample) is used to evaluate the predicted value of PRS methods. The following traditional PRS approaches are discussed in this review: (i) weighted methods that consider the effect sizes derived from GWAS result; (ii) unweighted methods that consider the single marker analysis; (iii) shrinkage methods that consider multivariate analysis. This review focuses on the tools and methods that perform PRS analysis and their applications in understanding the predictive power of PRS analysis. The reviewed PRS tools are chosen based on the following criteria:

1. The approach must perform PRS analysis based on “base” (GWAS) data (summary statistics) and “target” data set (genotypes and phenotypes in each of the target data set),
2. The approach may involve linkage disequilibrium pruning, and
3. The method or approach should be readily available as a tool or package so that it can be executed on any data set.

**Figure 1.**
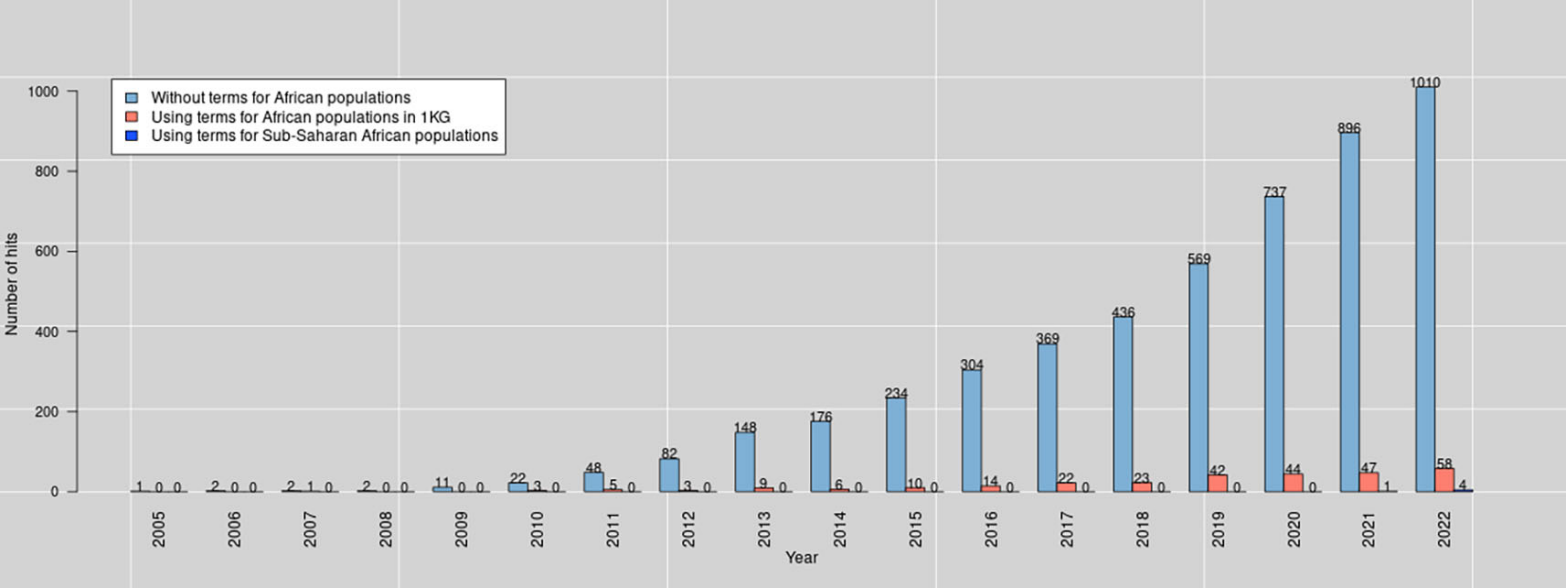
The number of PubMed hits per year (2005-2022) was obtained on December *23*, 2022, using query terms for PRS and African populations.

Besides reviewing PRS methods, we aim to investigate the application of PRS analysis in the sub-Saharan African population. It is worth mentioning that the term “African population” covers all those whose ancestors are Africans (including Africans in diaspora). Nevertheless, in this manuscript, the focus is on sub-Saharan Africa. When we searched PubMed for PRS publications in December *23*, 2022, the query reported *4,389* hits in total (see Figure 1 and text Box 1 for the query terms). For this review, we included articles based on their underlying PRS methods.

Box 1. **Pubmed query terms.**

We used the following terms for querying Pubmed for PRS:

( (“Polygenic Risk score”) OR (“Polygenic score”) OR (“Genetic Risk Score”) OR ( (“Genetic Risk”) AND (“GRS”)))

• We included the terms for Genetic Risk Score as some articles used them to refer to PRS.

We used the following terms for querying Pubmed for PRS for Africans:

( (“Polygenic Risk score”) OR (“Polygenic score”) OR (“Genetic Risk Score”) OR ( (“Genetic Risk”) AND (“GRS”))

AND

((African) OR (Africa) OR ((Yoruba) AND (YRI)) OR ((Luhya) AND (LWK)) OR ((Mandinka) AND (MAG)) OR ((Mende) AND (MSL)) OR ((Esan) AND (ESN))))

• For African populations (in red color), we included terms for Africans tribes based on 1,000 genomes.

We used the following terms for querying Pubmed for PRS for Sub-Sahran Africans:

( (“Polygenic Risk score”) OR (“Polygenic score”) OR (“Genetic Risk Score”) OR ( (“Genetic Risk”) AND (“GRS”))

AND

((subsahara) OR (“sub-saharan”)))

• The terms for sub-Saharan African populations are in red color.

Refer to Ref. 16, for the query syntax.

## Classification of PRS methods

**Figure 2.**
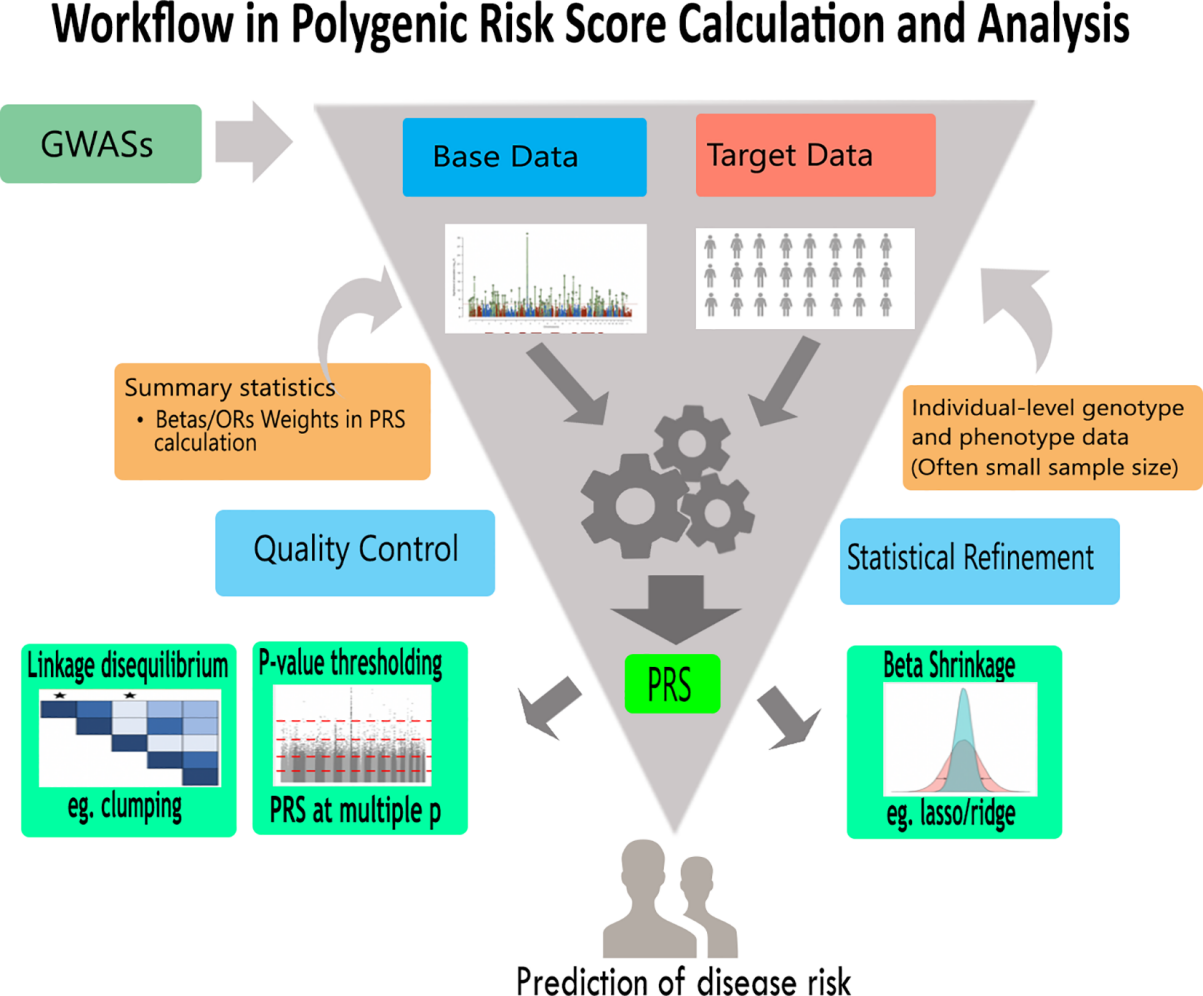
A general PRS analysis workflow. This is a typical polygenic risk score analysis workflow showing base data, target data and encapsulating different approaches. Using genotype and phenotype data,individual-level or summary statistics, approaches such as lasso/ridge regression, clumping and *p*-value thresholding can be employed to increase the predictive accuracy of PRS analysis. Furthermore, the results may be used to predict health or disease risk as well as give information for appropriate therapeutic approaches.

The different conventional approaches under the umbrella of PRS analysis are presented in Figure 2 and Table 1. We can categorize PRS methods into two; Bayesian-based and non-Bayesian methods. PRS methods can also be classified using their usage of linkage disequilibrium (LD): PRS methods that incorporate LD and PRS methods which apply LD pruning. To ease the understanding of their underlying algorithms, we grouped the PRS analysis approaches into four (see Table 2). Those with;

1. Clumping with thresholding (C + T)
2. *p*-value thresholding
3. Penalized regression
4. Bayesian shrinkage

**Table 1.** Summary of polygenic risk score tools. For more details refer to Ref. 37.

Tool	Approach	Computational platform	User friendly	Functionality
LDpred ^13^	Bayesian Shrinkage Prior	Python	Difficult	Uses a prior on effect sizes and LD information from an external reference panel
PRS-CS ^25^	Bayesian regression framework	Python	Difficult	Utilizes a high- dimensional Bayesian regression framework, by placing a continuous shrinkage (CS) prior on SNP effect sizes
EB-PRS ^20^	Empirical Bayes approach	R	Difficult	A novel method that leverages information for effect sizes across all the markers
AnnoPred ^21^	Bayesian Shrinkage Prior	Python	Difficult	A framework that leverages diverse types of genomic and epigenomic functional annotations
PRSice ^38^	Clumping + thresholding (C+T)	R	Difficult	For calculating, applying, evaluating and plotting the results of PRS analysis
PRSice2 ^39^	Clumping + thresholding (C+T)	C++, R	Easy	An efficient and scalable software program for automating and simplifying PRS analyses on large-scale data
LDpred2 ^40^	Bayesian Shrinkage	R	Difficult	A faster and more robust implementation of LDpred in R package bigsnpr
BSLMM ^41^	Bayesian sparse linear mixed model	R	Difficult	Prior specification for the hyper-parameters and a novel Markov chain Monte Carlo algorithm for posterior inference
BayesR ^24^	Hierarchical Bayesian Mixture Model	Fortran	Difficult	Bayesian mixture model that simultaneously allows variant discovery, estimation of genetic variance explained by all variants.
DPR software ^42^	Latent Dirichlet process regression model	C++	Easy	Dirichlet process regression to flexibly and adaptively model the effect size distribution.
SMTpred ^43^		Python	Difficult	Combines SNP effects or individual scores from multiple traits according to their sample size, SNP-heritability ( $h^{2}$ ) and genetic correlation ( $r_{G}$ ).
Lassosum ^22^	Penalised Regression	R	Difficult	A method for constructing PGS using summary statistics and a reference panel in a penalized regression framework.
Plink ^44^	*p*-value thresholding approach	C/C++	Easy	Open-source C/C++ toolset for GWAS analysis and research in population genetics.

**Table 2.** Comparison of different approaches for performing PRS analyses.

Key factors	Approaches			
	*p*-value thresholding with clumping	Penalised regression	Clumping + thresholding (C+T)	Bayesian shrinkage prior
Controlling for Linkage Disequilibrium	N/A	LD matrix is integral to algorithm	Clumping	Shrink effect sizes with respect to LD
Shrinkage of GWAS effect size estimates	*P*-value threshold	LASSO, Elastic Net, penalty parameters Bayesian	P-value threshold standard	Prior distribution, e.g. fraction of causal SNPs

### PRS methods that incorporate LD

In practice, When the markers are LD pruned, the prediction accuracy of PRS analysis tends to improve. Thus, the absence of LD information limits the predictive accuracy of PRS analysis. ^17^ For instance, the method of LD pruning and *p*-value thresholding (P + T) is commonly used, in the presence of LD patterns to improve the PRS prediction accuracy. ^13^ For instance, LDPred is a Bayesian approach that applies LD information in the presence of LD patterns. From this approach, the posterior mean effects of LD linked loci may be calculated analytically using a Gaussian infinitesimal prior, a non-infinitesimal model, in which only a portion of the markers is causative is perhaps a more realistic prior for effect sizes. For this reason, the following Gaussian mixture prior is considered:

$$\beta ∼\mathrm{iid}\left\{\begin{array}{ll} N\left(0,\frac{h_{g}^{n}}{M_{p}}\right) & \text{with probability}\;p\\ 0 & \text{with probability}\;\left(1‐p\right) \end{array},\right.$$ (1)

where p refers to the marker’s probability as the proportion of causal marker based on the Gaussian distribution. Similarly, the posterior mean in this model can be estimated using the equation below:

$$E\left(\frac{\beta_{i}}{{\tilde{\beta }}^{l}},D\right)\approx {\left(\frac{M}{{\mathrm{Nh}}_{g}^{2}}I+D_{i}\right)}^{-1}{\tilde{\beta }}^{l},$$ (2)

The LD matrix within the LD region is denoted by $D_{i}$ and the estimated effects within the target region are represented by ${\tilde{\beta }}^{l}$ , which is estimated using the least-squares method. The approximation assumes that the heritability explained by the region is small and LD with SNPs outside of the region is negligible.

### PRS methods that apply LD pruning

These PRS methods are non-Bayesian approaches that apply informed LD pruning (LD clumping) in PRS computation ( Figure 2). Generally, they are known as pruning and thresholding (P+T) methods. We may apply *p*-value thresholding, for example, with a univariate regression coefficient ( $r^{2}$ ) and a threshold of 0.2. To achieve prediction accuracy in the validation data, we would ensure that the *p*-value thresholding method is optimized across a grid. LD pruning, in which the less significant marker is pruned first, may result in more accurate predictions than random marker pruning. For the *p*-value threshold selection, researchers should include only SNPs that are statistically significant in GWAS. This technique essentially shrinks all omitted SNPs to zero estimates and does not perform shrinkage on the effect size estimates of the included SNPs. The optimal *p*-value threshold is a priori unknown and the targeted phenotype is assessed for the chosen threshold, which is why PRS is commonly computed over several thresholds. This technique can be interpreted as a variable selection process that essentially executes the GWAS *p*-value forward selection based on the size of the increment in the *p*-value thresholds.

### Bayesian approach in PRS analysis

Bayesian techniques have been successfully applied to model pre-existing genetic architecture with a prior that accounts for the range of effect sizes and thus increases polygenic score accuracy. The Bayesian statistical approach computes a refined posterior distribution from prior probability distributions using available data such as functional annotations. It shrinks marker effects by using LD information from a reference panel. ^18^ The key benefit of Bayesian-based PRS analysis is its ability to enhance PRS prediction accuracy from summary statistics by taking LD among markers into consideration. ^19^ Bayesian approaches in PRS explicitly model pre-existing genetic architecture that accounts for the distribution of effect sizes. These approaches allow the introduction of prior probability that improves the prediction accuracy of a polygenic score.

#### Empirical Bayes PRS (EB-PRS) method

The EB-PRS technique is an innovative method that relies on the Empirical Bayes theorem. It incorporates information across markers to strengthen prediction accuracy. ^20^ By utilizing the predicted distribution of effect sizes, the EB-PRS technique tries to reduce prediction error. Suppose all the SNPs are independent, the optimum PRS value is given by:

$$S=\beta^{T}X=\sum_{i=1}^{m}\beta_{i}X_{i},$$ (3)

where m denotes to the number of the all genotyped SNPs. The matrix $X_{i}$ stands for the genotypic value and $\beta_{i}$ is the log-odds ratio (OR) of the i th variant. The equation below can be used to measure the log-OR:

$$\beta_{i}=\log\left(\frac{f_{i1}\left(1-f_{i0}\right)}{f_{i0}\left(1-f_{i1}\right)}\right),$$ (4)

where $f_{i0}$ denotes the reference allele frequencies among the control samples and $f_{i1}$ denotes the reference allele frequencies among the target. If $\beta_{i}=0$ , that means the SNP is not correlated with the phenotype.

The actual values of effect sizes are generally unknown, thus they can be estimated empirically. Song *et al.* ^20^ used the Empirical Bayes method to estimate β . The estimators can be equally derived from GWAS summary statistics. Unlike other improved genetic risk prediction methods which utilize effect size distributions for PRS computation, the EB-PRS does not require external panels. ^13^^,^^19^^,^^21^^,^^22^ Also, the EB-PRS approach has theoretical superiority, resulting in a better PRS by lowering prediction error. The EB-PRS has recorded excellent performance in comparable to the other tool from following complex traits; Crohn’s disease, celiac disease, Parkinson’s disease, asthma, breast cancer, and type 2 diabetes. ^20^ Furthermore, a significant improvement was recorded when tested against the unadjusted PRS method, P + T, LDpred-inf, LDpred. ^19^ Although The EB-PRS approach has demonstrated that it can generate superior results without adjusting any parameters or relying on external data, studies have shown that further improvement is possible with a reference panel. For instance, the LD information as used in LDpred. Also, to increase the prediction accuracy, Song *et al.* ^20^ suggested that other available datasets such as GWAS summary statistics focused on functional annotations and genetically correlated traits could further improve EB-PRS accuracy.

#### Polygenic Risk Score-Continuous Shrinkage (PRS-CS) method

The PRS-CS is based on a Bayesian high-dimensional regression framework for polygenic modeling and prediction:

$$Y_{N\times 1}=X_{N}\beta_{M\times 1}+\varepsilon_{N\times 1},$$ (5)

where N refers to the sample size and M denotes the total number of the genetic markers. Y represents a vector of phenotypes/traits and X represents the genotype matrix. β is a vector of effect sizes for the genetic markers and ε is a vector of residuals. By assigning appropriate priors on the regression coefficients β to impose regularization, the additive PRS value can be calculated using a posterior mean effect sizes. LDpred ^13^ and the normal mixture model ^23^^,^^24^ have incorporated genome-wide markers with varying genetic architectures. The PRS-CS method aims to utilize a Bayesian regression framework and places a conceptually different class of priors (the continuous shrinkage (CS) priors) on SNP effect sizes. ^25^ On the other hand, continuous shrinkage priors allow for marker-specific adaptive shrinkage. The amount of shrinkage applied to each genetic marker is adaptive to the strength of its associative signal in GWAS, which accommodates diverse underlying genetic architectures. Ge *et al.* ^25^ presented the PRS-CS-auto method, a fully Bayesian approach that enables automatic learning of a tuning parameter ϕ , from GWAS summary statistics. Although analyses conducted from the Biobank indicate that for many disease phenotypes, the current GWAS sample sizes may not be large enough to accurately learn ϕ and the prediction accuracy of the PRS-CS-auto method may be lower than PRS-CS and LDpred. Nevertheless, simulation studies and quantitative trait analyses suggest that the PRS-CS-auto method can be useful when the size of the training dataset is large or when an independent validation set is difficult to acquire. Although the PRS-CS method provides a substantial improvement over the existing methods for polygenic prediction, ^13^ the current prediction accuracy of the PRS value is still lower than what can be considered clinical utility. Much work is needed to advance the predictive performance and translational value of PRS methods. Recent studies argued that jointly modeling multiple genetically correlated traits and functional annotations in polygenic modeling are expected to increase the predictive performance of PRS methods. ^26^^–^^28^

### PRS methods based on shrinkage of GWAS effect size estimates

Since SNP effects are calculated with uncertainty and not all SNPs have an impact on the traits, unadjusted effect size estimates of all SNPs can lead to a low-estimated PRS with high standards error. ^18^ Two shrinkage methods have been implemented to solve these problems; shrinkage of the effect estimates of all SNPs by adapted statistical techniques and use of *p*-value filtering thresholds as the criterion for inclusion of SNPs.

**Shrinkage of the effect estimates of all SNPs by adapted statistical techniques:** Some PRS methods performs shrinkage of all SNPs. These methods are typically apply shrinkage/regularisation techniques such as LASSO/ridge regression ^29^ or Bayesian approaches performing shrinkages by prior distribution specification. ^13^ Varying degrees of shrinkage may be accomplished under different methods or parameter settings. The most suitable shrinkages to be implemented depends on the underlying mixture of distributions of null and true effect size. PRS estimation is usually tailored over several (tuning) parameters since the optimum shrinkage parameters are a priori unknown. For example, it includes a setting for a fraction of causal variant ^13^ in the case of LDpred.

**p-value filtering thresholds as the criterion for inclusion of SNPs:** In this process, the PRS includes significant SNPs with a P-value below a choosen threshold (e.g. *p*-value < 23e-05). This method shrinks all omitted SNPs to an estimated effect size of zero and does not perform shrinkage on the effect size estimates of the included SNPs. Since the optimum *p*-value threshold is a priori unknown, PRS is computed over a range of thresholds associated with each of the tested target traits and optimized appropriately for the prediction. This is similar to optimizing parameters in the systematic shrinkage approach and regarded as a parsimonious method of variable selection. It is efficient in performing the forward selection of variables (SNPs) using GWAS and p-value with the sizes depending on the *p*-value threshold increment. Therefore, this forward selection method is the chosen’optimal threshold’. Furthermore, PRS derived from another subset of the SNPs may be more predictive of the target trait. Considering the fact that GWAS focuses on millions of SNPs, the number of subsets of SNPs for the study could be too large.

### Linkage disequilibrium control

Usually, association studies in GWAS are done individually. ^18^ The power of GWAS can be enhanced by leveraging the results of several SNPs concurrently. ^30^ Unfortunately, the raw data of all samples are not readily available. Researchers may need to take advantage of standard GWAS by considering either (i) SNPs are clumped such that the retained SNPs are almost independent of each other or (ii) all SNPs are included and the LD between them is adjusted. In the’standard’ polygenic scoring approach, option *i* is usually preferred and requires *p*-value thresholding. Option *ii* is commonly used in methods that incorporate conventional methods of shrinkage ^13^^,^^22^ (see Table 2). As for option *i* without clumping, some researchers tend to apply the methods of *p*-value thresholding. Although breaking this presumption can lead to marginal losses in certain situations. ^22^ Choi *et al.* ^18^ suggested that clumping should be applied when GWAS estimates of non-shrunk effect sizes are available. The standard method tends to work when compared to more advanced approaches. ^13^^,^^22^ It is possible that the clumping method captures conditionally independent effects. A critique of clumping for SNPs elimination in LD is that researchers usually use an arbitrarily selected correlation threshold. ^31^ Thus, no technique is without arbitrary features. This could be an area for the potential development of the classical method.

### PRS approach based on clustering and decomposition of genetic variants

PRS based variant decomposition focuses on decomposing or factorizing suitable genetic variants matrix into different components. This approach is mainly based on the use of an appropriate matrix decomposition technique. Contrary to traditional methods that compute PRS for a trait as the sum of effects from several genetic variants, this technique uses genetic risk for a single component to approximate risk for a weighted combination of relevant traits. Although there are many approaches to genetic variants decomposition, ^32^^–^^34^ only truncated singular value decomposition (TSVD) and singular value decomposition (SVD) have been used in the context of PRS.

Aguirre *et al.* ^35^ and Chasman *et al.* ^36^ are the first to use genetic risk decomposition to derive polygenic scores. They both applied TSVD and SVD respectively to compute polygenic risk scores from genetic components. While it is similar to the traditional PRS in predictive ability, it also enables an appropriate assessment of drivers of genetic risk for the phenotype. For example, Aguirre *et al.* ^35^ applied this method to body mass index and classified polygenic risk factors into overall health indicators, including sleep duration, alcohol, water intake, fat mass, fat-free mass. Consequently, they encouraged modeling PRS from the components of the decomposition of genetic risk association.

Let $W_{n\times m}$ be a sparse matrix of genetic associations with n rows and m columns, then TSVD can be performed on W to identify different genetic components. The decomposition will lead to factors of three matrices which approximates W :

• A singular matrix for trait $U_{n\times c}$ ,
• A singular matrix for variant $V_{m\times c}$ , and
• A diagonal matrix $S_{c\times c}$ of singular values. i.e., W .

Using the individual-level genotype vector $G_{m\times 1}$ , component polygenic risk scores (cPRS) can be computed by applying matrices U , S , and V , using the following formula

$$\text{cPRS}\approx S_{i}*V^{T}*G$$ (6)

Finally, PRS can be defined by summing through the component PRS, using cPRS for each component, then;

$$\mathrm{PRS}=\sum_{i}U_{\mathrm{ij}}*{\text{cPRS}}_{i}$$ (7)

## PRS tools

The next section will provide examples of some PRS tools that are commonly used to perform PRS analysis.

### Linkage Disequilibrium Pred (LDpred)

This method estimates the posterior mean effect size of each marker of GWAS summary data using a priori effect sizes and LD information from an external reference panel. ^13^ In this process, the inner products are re-weighted and the test-sample genotypes are the posterior mean phenotype. The posterior mean phenotype is an optimum predictor under the model assumptions and a point-normal mixed distribution is used as the effect size prior, allowing for non-infinitesimal genetic structures. Heritability explained by the fraction of causative markers and genotypes are the two parameters of the prior. The heritability parameter is calculated using summary statistics from GWAS and takes into account sample noise and LD. ^45^

In an attempt to check the performance of LDpred in comparison to the method of pruning followed by thresholding, using five complex traits, including breast cancer, schizophrenia, muscular dystrophy, and coronary artery disease. GWAS summary statistics for large sample sizes ranging from 27,000 to 86,000 individuals and raw genotypes for an independent dataset validated, LDpred outperforms the other approach ^19^ particularly at large sample sizes. For instance, the predicted $R^{2}$ rose from 20.1 percent to 25.3% and from 9.8% to 12.0% in a large dataset of schizophrenia and multiple sclerosis, respectively. Although the accuracy of the predictive values were lower in absolute terms in another study to predict schizophrenia risk in non-European validation populations of African and Asian heritage, similar observations were made for other approaches.

LDpred is a powerful tool that can be used for performing polygenic scores using summary statistics and LD information. ^13^ However, one of its limitations is that its underlying algorithm assumes the existence of causal variants, which may result in limited predictive performance. In addition, its Gibbs sampler is sensitive to the model parameters for the large sample sizes. Moreover, LDpred can not predict PRS accurately for genomic regions with long-range LD, for instance, the human leukocyte antigen (HLA) region of Chromosome 6 _._^24^^,^^26^ However, long-range LD regions of the genome might contain many known disease-relevant variants. ^46^^,^^47^ Privé *et al.* developed a new version of LDpred to address these shortcomings and improve its computational efficiency. ^40^ This new version of LDpred has been implemented in the R package bigsnpr; see the next section.

### LDpred2

LDpred2 is the improved version of LDpred tool by introducing new options to learn the effect accurately. For instance, the option *sparse* can estimate the effects that are 0 while the option *auto* can estimate the parameters from data and computes values for hyper-parameters p and $h^{2}$ . Due to these improvements, LDpred2 has been widely used to generate polygenic models with good predictive performance. ^48^ However, LDpred2 still has some issues regarding its stability. ^24^^,^^26^ These issues contributed to the discrepancies in reported prediction accuracies. ^39^^,^^49^ For instance, in contrast to LDpred, LDpred2 performs very well in the HLA regions but not for all traits as LDpred2 does not perform well for type 1 diabetes (T1D) and pure red cell aplasia (PRCA). LDpred2 performs poorly on T1D because T1D is mainly composed of large effects in the HLA region, while summary statistics typically have a small sample size. However, it is unknown why LDpred2 performs poorly, specifically for PRCA. Further studies are needed to understand why LDpred2 under-perform in these two cases.

### PRSice

PRSice, developed by Euesden *et al.* ^38^ in 2015, was the first specialized PRS analysis program. PRSice is built in R and includes wrappers for bash data management scripts as well as PLINK-1.9 to speed up computation ( Table 1). Using a list of m SNPs and n individuals from the ‘target phenotypic’ dataset, here, thegenotypes have some influence on the ‘base phenotype’. If assessing the common genetic overlap of phenotype between samples/populations, the base and target phenotypes may be the same. A univariate regression on the base phenotype for each SNP, such as from genome-wide association research, can be used to estimate genotype effects (GWAS). For a SNP i , where i = 1, 2, …, m , a *p*-value, $P_{i}$ , is computed for the association between the SNP and genotypes, $G_{i}$ , $j=\left\{0,1,2\right\}$ for individual j where j = 1, 2, …, n and the phenotype. Under the standard additive assumption used in GWAS, a corresponding effect size for the effect of a unit increase in genotype Gij on the phenotype is estimated by $\beta_{i}$ . The degree of estimate is used to determine which SNPs should be included in a PRS value. SNP i will be included in in a PRS computation if Pi is less than a threshold, $P_{T}$ , based on the *p*-value for their association with the base phenotype in a GWAS. Typically, PRS values are calculated at distinct $P_{T}$ *p*-value thresholds.

At threshold $P_{T}$ , the PRS value for individual j can be calculated as:

$${\mathrm{PRS}}_{\mathrm{PT},j}=\sum_{i=1}^{m}\beta_{i}G_{i,j}.$$ (8)

The PRS value is computed across all individuals, yielding n scores per $P_{T}$ threshold value. A suitable regression model could be used to assess the relationship between these PRS values and the target phenotype. The PRSice tool was created to fully automate PRS analyses, significantly enhancing PLINK-1.9’s capabilities. ^50^ Unless the genotypes have previously been imputed, there is generally some missing genotype data in real data. PLINK-1.9 fills in any missing data using mean allele frequencies. Nevertheless, it is not equipped to handle very large data sets. Hence a more memory-efficient approach is used in its advanced version, PRSice-2.

### PRSice-2

PRSice-2 is an improved version of PRSice. It works with genotyped and imputed data, gives empirical association *p*-values that are free of overfitting inflation, supports numerous inheritance models, and analyzes numerous continuous and binary target traits at the same time. ^39^ This technique simplifies the PRS analysis pipeline by eliminating intermediary files and doing all of the core computations in C++, resulting in a significant decrease in execution time and memory use. Furthermore, while computing the PRS value, PRSice-2 can immediately handle the BGEN imputed format and convert it to either best-guess genotypes or doses without producing a big intermediate file. While PRS values based on best-guess genotypes are produced using genotyped input, PRS values based on dose are derived using the following formula:

$$\mathrm{PRS}=\sum_{i}^{m}\beta_{i}\left(\sum_{j}^{2}\omega_{\mathrm{ij}}X_{j}\right).$$ (9)

Where $\omega_{\mathrm{ij}}$ is the probability of observing variant j , the value of $j\in \left\{0,1,2\right\}$ , for the $i^{\mathrm{th}}$ SNP/variant; m represents the number of SNPs/variants; and $\beta_{i}$ denotes the effect size of the $i^{\mathrm{th}}$ variant estimated from the relevant base data set. A simulation study has been used to compare the performance of PRSice-2 to alternative polygenic score software lassosum ^22^ and LDpred ^13^ in terms of run time, memory usage and predictive power on servers equipped with 286 Intel 8168 24 core processors at 2.7 GHz and 192 GB of RAM.

Based on a simulation study, PRSice-2 outperformed lassosum and LDpred in all circumstances. PRSice-2, in particular, can do full PRS analysis on 100,000 samples in 4 minutes, 179 times quicker than lassosum, which required 10 hours for the same task, and 241 times faster than LDpred, which took about 13 hours 27 minutes. Similarly, PRSice-2 uses substantially less memory than lassosum and LDpred, requiring less than 500 MB for 100,000 samples against 11.2 GB for lassosum and 45.2 GB for LDpred.

In another study to compare its predictive power for quantitative traits with a heritability of 0.2 and a base sample size of 50,000, and a target sample size of 10,000, PRSice-2 resulted in PRS values that are higher than LDpred but not as high as lassosum. The details about how it performs, inspection and analyses can be found ( here). While the PRS values obtained by PRSice-2 do not fully optimize prediction accuracy, the straightforward technique and use of fewer SNPs allow for a clearer understanding of the results when compared to approaches that employ all SNPs. ^51^

### Lassosum

Lassosum is an alternative method that uses summary statistical data to estimate PRS and takes LD into account by using reference panels ^22^ based on the commonly used LASSO and elastic net regression. ^52^^,^^53^ Consider the linear regression given below:

y=Xβ+ε. (10)

For which X represents a data matrix of *n*-by- *p*, and y denotes a vector of the observed outcome. LASSO is a commonly used method for deriving β estimates and y predictors, especially in cases where p is high and where it is rational to conclude that many β are 0. By minimizing the objective function, LASSO also obtains estimates of β given y and X . To test the efficiency of lassosum relative to LDpred, simulation studies were carried out using summary statistics accounting LD and Phase 1 data from Welcome Trust Case Control Consortium (WTCCC) for seven diseases. ^13^ The outcome of LDpred, lassosum and simple soft-thresholding (setting s = 1 in lassosum) was compared with most of the diseases in the WTCCC dataset, except for T1D where lassosum seem to outperform LDPred. The performance of LDpred and lassosum was comparable when the number of causal SNPs was 1,000 and the sample size was 11,200 for the simulated phenotypes, and both were superior to soft thresholding. Unlike lassosum, LDpred’s performance was considerably reduced when the sample size was halved. The lassosum was not influenced in the same way when reducing the sample size by half. All methods performed equally when the number of causal SNPs was 25,000 and the sample size was 11,200. The fact that summary statistics can be confounded by population stratification and population heterogeneity makes the real-life application of PRS difficult. These problems in the lassosum design were not considered. One possible issue with the use of meta-analytical summary statistics is that the original data produced by the summary statistics was an amalgamation of datasets around the world with corrections for population stratification. There is possibly no homogenous dataset suitable as a reference panel. Further research is required to explain the best approach.

Schork *et al.* ^54^ have demonstrated that different genome regions have different false discovery rates, thus have different chances of being causally correlated with a phenotype. Genome annotation information can be used theoretically to enhance the performance. Similarly, it is possible to utilize the fact that certain phenotypes have common genetic determinants (pleiotropy) to improve PRS.

### PLINK SOFTWARE (Second-generation PLINK)

PLINK 1 is an open-source C/C++ toolbox for population genetics research and GWAS data analysis. The increasing rise of data from imputation and whole-genome sequencing research necessitated the urgent need for speedier and scalable implementations of its essential functionalities. Furthermore, genotype likelihoods, phase information, and multiallelic variations are commonly found in GWAS and population-genetic data. However, these features cannot be handled by PLINK 1 primary data format cannot accommodate any of these. For these reasons, Chang *et al.* ^44^ developed a new version called PLINK 1.9. This version features heavy use of bit-level parallelism, O (pn)-time/constant-space Hardy-Weinberg equilibrium computation, Fisher’s exact testing, and a slew of other algorithmic enhancements. PLINK 1.9 speeds up most processes by 1-4 order of magnitude, allowing it to handle data sets that are too huge to store in RAM. The basic functional domains of PLINK 1.9 are identical to those of its predecessor, and it may be used as a drop-in replacement for existing scripts in most circumstances. Features, including the import/export of VCF, Oxford-format files, and fast cross-platform genomic relationship matrix calculators, have been included to facilitate easier interoperability with newer applications. Despite its computational advantages, PLINK 1.9 may still be an unsuitable tool for working with imputed genomic data due to the limitations of the PLINK 1 binary file format. To address this problem, the authors have developed PLINK 2.0, which features a new core file format capable of holding the bulk of the data generated by modern imputation systems.

### PRS tools in diverse populations

Applying PRS analysis for multi-ethnic groups is still limited. Novel PRS methods have been developed to address the applicability of PRS analysis across ethnic groups.

**Multi-ethnic PRS analysis:** Multi-ethnic PRS analysis is a new PRS approach that combines PRS analysis based on two distinct populations. ^55^ For instance, multi-ethnic PRS analysis could merge PRS analysis based on European training data with PRS analysis based on training data from another population. The multi-ethnic PRS approach computes PRS value given a target individual with genotypes g as follows:

$$\mathrm{PRS}=\sum_{i=1}^{M}{\hat{b}}_{i}g_{i},$$ (11)

where M denotes the number of individual’s genetic markers, and the term ${\hat{b}}_{i}$ is an estimate of effect sizes. For a multi-ethnic PRS analysis, this approach uses a linear combination of the two distinct PRS values and applying mixing weights parameters $\alpha_{i}$ .

**Linear unbiased predictors (BLUP):** PRS analysis can be molded using the well-known approach of best linear unbiased predictors (BLUP). ^56^ BLUP is used to consider and linearly model both random effects and fixed effects. It is also known as genomic best linear unbiased prediction (gBLUP). ^57^ BLUP/gBLUP estimates PRS values using the following formula

PRS=Xβ+g+ε, (13)

Where β represents a vector of the fixed effects, g denotes the total genetic effects in the base/training dataset, and ε are the normally distributed residuals. To evaluate the fixed effects, BLUP considers an individual GWAS indicator, the top 5 principal components (PCs) derived with all samples together and/or a list of the significant SNPs. The BLUP approach is a computationally efficient algorithm. Nevertheless, the limitation of BLUP arose due to its requirement of the Individual-level genotype data. BLUP has been implemented in GCTA software (Genome-wide Complex Trait Analysis) . Moreover, it has been extended to XP-BLUP to model PRS values for admixed populations. ^57^ Also, BLUP has been extended to MultiBLUP to include multiple random effects. ^58^

**Genetic Risk Scores Inference (GeRSI):** GeRSI uses mixed models by combining fixed-effects models and random-effects models for controlling population structure. ^59^ GeRSI performs Gibbs sampling to estimate individuals’ genetic risk score given the case-control study’s genotypes under a random-effects model. GeRSI proposed conditional distributions of the genetic and environmental effect using the standard liability-threshold model. One limitation of GeRSI is that it requires individual-level genotypes which are not available to many bioinformaticians.

**Cross-population BLUP (XP-BLUP):** XP-BLUP is an extension of the BLUP method that can be applied to trans-ethnic populations. ^59^ XP-BLUP utilizes trans-ethnic information to improve PRS value predictive accuracy in minority populations. It combines the linear mixed-effects model (LMM) of the GeRSI method with the BLUP method.

**PRS-CSx:** PRS-CSx method is expected to improve the accuracy of the application of PRS across multi-ethnic populations by using posterior inference algorithm. ^60^^,^^61^ PRS-CSx combines GWAS summary files from different population to increase the accuracy of PRS. PRS-CSx estimates population-specific effect size by incorporating the population-specific LD pattern, population-specific allele frequency information and the information of shared continuous shrinkage prior across populations. For more details about the mathematical method underlying PRS-CSx, refer to Ref. 60.

## PRS analysis and population structure

The main cause of false-positive genotype-phenotype associations in PRS analysis is from population genetic structure. ^18^^,^^62^ In African populations with population structure, GWAS analysis techniques provide a significant rate of false-positive results. ^63^ These findings are influenced by the cohort’s relatedness rather than variations that have an effect on the trait or disease risk. ^63^ In general, structures in mating patterns induce structures in genetic variation closely associated with geographic location. Furthermore, risk factors due to the environmental exposure may be creating the possibility for correlations between genetic variations. Sul *et al.* ^63^ have noted some confounding issues that are unique to GWAS research, such as 1) genetic artifacts such as errors on SNP array chips; 2) phenotypic and environmental diversity in the participants, such as gender, ancestry, and age; and 3) strategic ignorance about disease risk. ^62^ These confounding factors affect the genomic composition of populations and are difficult to calculate as they are not openly evident. ^18^^,^^62^^,^^63^ The characteristics examined are confounded by example and location. ^64^^,^^65^ Usually, this issue is resolved in GWAS by modifying the PCs ^64^ or by using mixed models. ^66^

The population composition in the PRS study presents a possible great issue since there are a significant number of null variants in PRS estimation. For example, allele frequencies are systematically different between the base and target data. These can be obtained from genetic drift or genotyped variants. ^67^ In addition, there is a danger that variations in null SNPs may result in the correlation between the PRS and target traits if the distributions of the environmental risk factors for the phenotype vary in base and target data or highly probable in most PRS studies. Even if the GWAS had completely regulated its population structure, confounding is possibly reintroduced. Correlated variations between the base and target data in allele frequencies and risk factors are not taken into consideration.

The regulation of structure in the PRS study should be adequate to prevent false-positives, if the base and target samples are drawn from the same or genetically similar populations. Choi *et al.* ^18^ advised that there are drastic variations between populations in the distribution of PRS. ^67^^–^^69^ Such observations do not indicate many differences between populations in etiology. Genuine differences are likely to contribute to geographical, cultural and selection pressure variations. It challenges the use of base and target data from different populations in PRS studies that do not tackle problems of possible uncertainty generated by geographical stratification. ^68^ Therefore, by exploiting large sampling sizes, the effect can be obtained using subtle confounding. The issues of population structures are as important as the variations between individuals in the base and target populations in genetics and the environment. In the coming years, the discussion of generalizability of PRS methods across populations can be an active field. ^55^^,^^69^

## Population bias in available genotyping platforms

The PRS method that could be applied to diverse populations is still a challenging task. ^68^ Many factors limit the application of PRS across diverse populations. These factors include:

• The limitation in the current genomics technologies
• LD distribution across diverse population
• The minor allele frequencies (MAF) distribution
• The distribution of the causal variants across diverse populations.

Current sequencing technologies are based on the European reference genome. Hence, the current genomics technologies are still not robust enough to capture genetic diversity among trans-ethnic populations. Studying LD patterns across diverse populations showed that the distribution of LD patterns plays a critical role in the underlying PRS value. ^70^^,^^71^ Incorporating the information of LD patterns across diverse populations would increase PRS utilities among trans-ethnic populations. Moreover, the utility of PRS across diverse populations has limited the MAF across diverse populations. ^68^^,^^70^ The differences in MAF variants across diverse populations will result in different variant selection, ^72^ which will reflect PRS in calculations. Furthermore, to improve the utility of PRS across diverse populations, researchers should investigate the causal variants shared across multi-ethnic groups. ^73^ Type 2 diabetes and body mass index account for 70-80% of African ancestry. However, because of variations in LD and allele frequency, the accuracy of African-based PRS was lower than that of European-based PRS. Some studies showed that Europeans’ causal variants are also likely to be shared in African ancestry. ^74^^–^^76^ Despite this, we can not generalize that the causal variants shared among trans-ethnic groups due to the limitation of representation of non-European populations, including sub-Saharan African communities. Previous approaches introduced to increase PRS accuracy in African populations prioritize the use of population-specific weighting and European discovered variants. However, due to the small sample sizes in African population, only moderate gains in accuracy are attainable. The example of a method that allows ethnic-specific weights to be included in their model is a two-component linear mixed model. In another study, Márquez-Luna *et al.* ^55^ used Latino training data with limited sample size and publicly available large sample size European summary statistics to predict type 2 diabetes in a Latino cohort. When compared to previous methodologies, they achieved a relative improvement in prediction accuracy of more than 70%. This technique was also used to predict height using European and African training data in an African UK Bio bank.

## Limitations of current PRS algorithms

The methods for performing PRS vary based on two primary factors: (i) the list of SNPs to be used, and (ii) the weights to be used. Given the LD structure between SNPs, depending on the the trait’s genetic architecture and GWAS discovery sample size, the appropriate technique for determining what weights to apply and which SNPs to choose will differ between traits. The following tools LDpred, LDpred funct, SBLUP, P+T, LDpred-Inf PRS-CS, SBayesR, and PRS-CS-auto were employed in a comparative study to assess the PRS approaches in terms of their predictive potential. ^77^ To accomplish this task, data from the major depressive disorder and Psychiatric Genomics Consortium working groups on schizophrenia were used. The results demonstrate that SBayesR outperforms the other tools in terms of speed and predicted accuracy. SBayesR, on the other hand, cannot produce converged solutions if the GWAS summary statistics have non-ideal features. While the benchmark P+T approach performed the least, the other tools achieved nearly the same level of accuracy. In addition to being the best approach in this study, SBayesR has been designed to learn the genomic architecture from the GWAS attributes. Some of these approaches, including LDpred, use tuning cohorts to specify parameters for the target cohort. When the length of the Markov chain Monte Carlo chain increases for example in LDpred, the prediction accuracy improves. One drawback of such strategy is that the user will have to tune the model parameters. Substantial effort is currently ongoing to expand GWAS sample collection across demographic groups. Most of the existing tools use only samples of European ancestry in the comparative PRS study. As a result, further study is needed to assess the accuracy of alternative techniques in other ancestries and across ancestries, taking into account probable differences in genomic architectures and LD.

## The predictive power of PRS analysis

Most articles within the current literature consider sample size as a milestone to power the PRS analysis. In 2013, Dudbridge estimated the predictive power of the polygenic score using results from several published studies. ^12^ Dudbridge concluded that all published studies with a significant association of PRS values are statistically well-powered. In addition, Dudbridge pointed out that the accuracy of the PRS analysis depends only on the size of the initial data set (training sample). Furthermore, he provided a mathematical model to estimate the statistical power of PRS value as a function of sample size. In 2014, Middeldorp *et al.* ^29^ suggested that PRS analysis on a sample size of 2000 individuals is good enough to obtain a statistically powered PRS value. However, Dima and Breen in 2015 ^78^ demonstrated that a sample size of 1500 is enough to increase the predictive power to a statistically significant point. They stated that the predictive power of polygenic risk scores is not good enough for clinical utilities but it could be used as a biomarker for traits of interest within individuals. Recently, in 2017, Krapohl *et al.* ^5^ introduced a multi-polygenic score that is capable of increasing the predictive power of PRS analysis. Regarding the relative accuracy of PRS values across ancestries, Yengo *et al.* ^79^ proposed a theoretical model to estimate them. Their method utilized the frequencies of the minor alleles (MAF) in the two populations, the LD between the causal SNPs and the heritabilities. The authors assumed that causal variants are shared across ancestries however, their effect sizes might vary. Based on their model, Yengo *et al.* ^79^ concluded that LD and MAF differences across ancestries explained 70-80% of the loss of relative accuracy of European-based PRS value in African ancestry.

Zhao & Zou (2022) showed in their study that PRS predictivity can be improved based on SNPs selection. The process of SNPs selection depends on the genetic architecture, i.e, causal variants, and the sample size of the training data set. ^80^ To select a set of SNPs that provide the optimal PRS prediction, the sample size of the training data set should be much larger than the number of potential causal variants. That is, performing PRS where the ratio of causal variants and sample size is large results in poor PRS prediction due to failure in causal variants separations. Therefore, in the case of the ratio of causal variants to the sample size is large, i.e., small sample size is the training data set, Zhao & Zou recommended that a large number of variants should be included to get higher PRS prediction power. They further recommended the addition of independent uncorrelated variants to improve PRS predictivity. Moreover, Zhao *et al.* (2022) demonstrated that accounting for correlation between causal variants, i.e., LD will improve PRS predictivity and accuracy for heterogeneous populations. ^81^ Furthermore, the performance of the PRS mathematical model can be assessed by evaluating the model’s output using machine learning techniques including area under the curve (AUC) of the receiver operating characteristic (ROC). ^82^^,^^83^ The ROC can be visualized by plotting true positive rate against false positive rate for model’s thresholds. Janssens *et al.* (2007) recommend using a model that provides AUC >0.75 for PRS clinical utility which involves the screening of individuals who are at risk. In addition, Igo *et al.* (2019) ^82^ has suggested using the proportion of trait variability explained by one or more variants as an indicator for PRS predictivity. For more details refer to Refs. 82, 83.

## PRS clinical utility

PRS analysis has been successfully applied to estimate and identify individuals with genetic risk for many biological traits such as type 2 diabetes, breast cancer, and prostate cancer (See the extended data ^122^). Most of these studies provide significant evidence of the success of PRS analysis in identifying patients who are at high risk of developing disease complications. Additionally, the primary strength of PRS analysis is its capability of stratifying individuals based on their probability of developing a disease. The biological power of PRS analysis arose due to its capacity to identify therapeutic and genomic pathways for type 2 diabetes, breast cancer, and prostate cancer. Moreover, applying PRS analysis on these traits showed that the reproducibility of PRS results is in the European population.

Nonetheless, one weakness of applying PRS analysis on these traits is its limited ability in detecting false-positive results. It is observed that most PRS studies are only available for European ancestries. Therefore, we can not apply them to non-European communities. In addition, performing PRS analysis on sizeable multi-ethnic data is indispensable for obtaining more accurate PRS values across populations. Furthermore, the possibility of applying PRS outcomes for personalized medicine requires robust validation procedures before broad clinical applications for multi-ethnic communities.

Understanding complex diseases and their clinical manifestations can be advanced significantly using accurate models for estimating PRS. The current PRS models can be used to forecast outcomes accurately. Disease subtypes and mechanisms that underpin within-trait diversity are not accounted for in PRS models, which might be important for analysis or therapeutic response. ^35^^,^^36^^,^^84^^,^^85^ PRS models are used mainly to estimate clinical risk prediction for certain diseases, that can be extended to lifetime risk trajectories. ^86^^,^^87^ Furthermore, PRS models can be implemented by clinical care authorities to decrease potential adverse health outcomes. Public health authorities can benefit from PRS models to control outbreaks of a particular disease by providing more efforts in high risk areas. PRS models can be used to define policies for administering the vaccination process. To use PRS accurately in clinical utilities as a personalized medicine tool, factors such as family history, rare monogenic mutations, ethnicity and ancestry, indirect genetic effects and gene-environment correlation should be considered. Refer to Table 3 for some commercial PRS kits that can be used for clinical utilities.

**Table 3.** Examples of PRS kits for clinical utilities.

Company	PRS Kit	Disease/Usage	Variants/Genes	Link
Illumina	Infinium Global Screening Array v3.0	Autoimmune disorders, childhood diseases, drug responses.	654,027	https://www.illumina.com/
	Infinium Global Screening Array with Multi-disease drop	Span of diseases: psychiatric, neurological, cancer, cardiometabolic, autoimmune, anthropometric.	≈ 50K variants	
	Neuro Array	Extensive neurodegenerative disease.	180K	
	Oncoarray	Disease markers for a wide range of tumor types.	499,170	
	DrugDev Consortium Array	Drugable targets.	485,000	
	H3Africa Consortium Array	Epidemiological research: Somatic mutations in cancer, Disease defense, transplant rejection, and autoimmune disorder, drug responses.	10,000	
	PsychArray	Common psychiatric disorders such as schizophrenia, attention deficit hyperactivity disorder, bipolar disorder, major depressive disorder, autism-spectrum disorders, obsessive-compulsive disorder, anorexia nervosa and Tourette’s syndrome.	≈ 30K	
23andMe	1- Health + Ancestry Service 2-23andMe + Membership	Several diseases, including breast cancer, diabetes, MUTYH-Associated Polyposis, Late-Onset Alzheimer’s Disease, Parkinson’s Disease, lung and liver disease, Chronic Kidney Disease, Familial Hypercholesterolemia, anemia, nerve and heart damage, and iron overload.	7,400-45,000 markers per chromosome	https://www.23andme.com/
Allelica	SCT-I	Chronic diseases, including coronary artery disease.	1920136	https://www.allelica.com/
Ambry Genetics	AmbryScore	Breast cancer.	100	https://www.ambrygen.com
Genetic Technologies	COVID-19 Severity Risk Test	COVID-19	Not Provided	https://www.globenewswire.com
	GeneType for Breast Cancer	Breast cancer.	77 loci for Caucasian women, 74 for African American women and 71 for Hispanic women.	
	GeneType for Colorectal Cancer	Colorectal cancer.	45	
Color	Hereditary Cancer Test	Cancers: uterine, pancreatic, ovarian, colon, melanoma, breast, stomach, and prostate cancers.	30 genes	https://www.color.com
	Hereditary Heart Health Test	Heart disease.	30 genes	
AnteBC	AnteBC – Breast Cancer Polygenic Risk Score Test	Breast cancer.	2803	https://antegenes.com/
Applied Biosystems	UK Biobank Axiom Array	Cancer common variants, Lung function phenotypes, Alzheimer’s disease.	246,055	https://www.thermofisher.com/

## PRS Analysis on sub-Saharan African populations

The PRS Analysis on sub-Saharan African populations is limited due to lack of enough GWAS studies on traits associated them. For instance, searches on PubMed for PRS on sub-Saharan African populations on December 23, 2022 (see Figure 1 and Box 1) resulted in only 5 hits ( *4* research articles and *1* review paper). The four research articles performed PRS analysis mainly on traits associated with cardiometabolic diseases such as heart attack, Type 2 diabetes, and stroke. Other contributing risk factors include body mass index (BMI), waist circumference (WC), hip circumference (HC), waist-to-hip ratio (WHR), systolic blood pressure (SBP), diastolic blood pressure (DBP), triglycerides (TG), total cholesterol (TC), low-density lipoprotein(LDL), high-density lipoprotein (HDL), fasting plasma glucose( FPG), and Type 2 diabetes (T2D), low-density lipoprotein cholesterol (LDL-C), high-density lipoprotein cholesterol (HDL-C), triglycerides (TGs) and total cholesterol (TC). ^88^^–^^91^ More so, the variance detected for sub-Saharan populations in these studies has been summarized in Table 4.

**Table 4.** Examples of the application of PRS studies that are conducted in sub-Saharan African populations.

Disease	Methods	Populations	LD reference panel	Trait	Variance detected for sub-Saharan R ^2^	p-value
Cardiometabolic traits ^1^ [ 88]	PLINK 1.9 weighted sum of the number of risk variants	sub-Saharan Africans (n = 5,200), African Americans (n = 9,139) and European Americans (n = 9,594)	1,000 Genomes (prunedGRS for independent variants)	BMI	0.0767	0.0001
				WC	0.5700	0.2749
				HC	0.5545	0.1898
				WHR	0.1965	0.7781
				SBP	0.1640	0.3068
				DBP	0.0659	0.0213
				TG	0.1803	2.83e-06
				TC	0.0628	6.89e-14
				LDL	0.0781	1.45e-19
				HDL	0.0403	5.44e-12
				FPG	0.0447	0.2788
				T2D	0.1180	6.84e-08
Cardiometabolic ^2^ [ 88]	PLINK 1.9 weighted sum of the number of risk variants	sub-Saharan Africans (n = 5,200), African Americans (n = 9,139) and European Americans (n = 9,594)	1,000 Genomes (prunedGRS for independent variants)	BMI	0.0741	0.0001
				WC	0.5700	0.2749
				HC	0.5545	0.1898
				WHR	0.1967	0.7781
				SBP	0.1640	0.3068
				DBP	0.0651	0.0213
				TG	0.1761	2.83e-06
				TC	0.0502	6.89e-14
				LDL	0.0596	1.45e-19
				HDL	0.0293	5.44e-12
				FPG	0.0447	0.2788
				T2D	0.1050	6.84e-08
Cardiometabolic ^3^ [ 89]	PRSice-2	African American (n = 61,796), European (n = 24,154), multi ancestry opulations (African American, European and Hispanic American) (n = 25,747), Zulu cohort (n = 2,598), Ugandan cohort (n = 6,407)	1,000 Genomes	HDL-C	0.0213	3.97e-15
				LDL-C	0.0814	6.83e-53
				TG	0.0087	8.97e-07
				TC	0.0693	4.43e-46
Cardiometabolic ^4^ [ 89]	PRSice-2	African American (n = 61,796), European (n = 24,154), multi ancestry populations (African American, European and Hispanic American) (n = 25,747), Zulu cohort (n = 2,598) Ugandan cohort (n = 6,407)	1,000 Genomes	HDL-C	0.00003	0.6432
				LDL-C	0.00026	0.1696
				TG	0.00002	07620
				TC	0.00048	0.0534
Heart failure ** [ 98]	-	-	-	-	-	-
Cardiometabolic ^5^ [ 90]	Clumping and thresholding (C+T) approach in PRSice2	Stage 1: (n = 10,603): AWI-Gen dataset from Eastern, Western and Southern Africa). Stage 2: (n = 23,718): AWI-Gen dataset + 4 cohort studies : Uganda Genome Resource, Africa-America Diabetes Mellitus, Durban Diabetes Study, and the Durban Case Control.	1- African Reference Panel at the Sanger Imputation facility 2- 1,000 Genomes	LDL-C	0.0675	1.10e-63
				HDL-C	0.0118	9.62e-11
				TG	0.0098	2.02e-17
				TC	0.0218	4.05e-20
Cardiometabolic ^6^ [ 90]	Clumping and thresholding (C+T) approach in PRSice2	Stage 1: (n = 10,603): AWI-Gen dataset from Eastern, Western and Southern Africa). Stage 2: (n = 23,718): AWI-Gen dataset + 4 cohort studies : Uganda Genome Resource, Africa-America Diabetes Mellitus, Durban Diabetes Study, and the Durban Case Control.	1- African Reference Panel at the Sanger Imputation facility 2-1,000 Genomes	LDL-C	0.0745	6.58e-131
				HDL-C	0.0117	2.80e-28
				TG	0.0098	2.02e-17
				TC	0.0303	9.93e-45
Adiponectin level [ 91]	Clumping and thresholding (C+T) approach using the PRSice-2	Unrelated sub-Saharan Africans (n = 3,354); 1- Africa America Diabetes Mellitus, 2- T2D cases from Nigeria, Ghana, and Kenya	Haplotype Reference Panel via the Sanger Imputation Service	Insulin resistance, HDL, LDL, total cholesterol, triglycerides, blood pressure, T2D, and hypertension.	The exact value is not given. However authors provided the adiponectin PRS with the best model fit as a figure	

^1^ Using GRS Model.

^2^ Model without GRS.

^3^ PRS on Zulu cohort.

^4^ PRS on Ugandan cohort.

^5^ PRS on 1/3rd of the AWI-Gen cohort as Test-set, 6-PRS on 2/3rd of the AWI-Gen cohort as Test-set.

^**^ It is a review article.

However, it recommended the usage of PRS on Heart Failure management.

The general outcome of these five articles emphasize an urgent need for GWAS research studies for sub-Saharan African populations in order to continue to perform PRS analysis that would add more benefits to the use of PRS in precision medicine as well as an improved representation of multiple ethnic populations in GWAS to better reflect risk stratification, variabilities in genetic equitable and translation of GRS in clinical setting. For instance, Ekoru *et al.* (2021) ^88^ demonstrated that several traits such as cardiometabolic have less predictive power of genetics risk score in sub-Saharan Africans compared to others populations such as African Americans and European Americans. The less predictive power of cardiometabolic traits was as a result of underrepresented African populations based on GWAS data in the current reference genomes. However, Kamiza *et al.* (2022) ^89^ studies showed an increase in PRS performance on lipid traits (such as, LDL-C) with dataset from sub-Saharan populations, European and multi-ancestry. Other lipid traits include HDL-C, TGs and TC. Kamiza *et al.* reported that PRS performance varies significantly even among the sub-Saharan African populations. This variation on PRS performance occurs due to variations on Africa population-specific genetic structure such as minor allele frequencies and the population-specific associated environmental factors. Moreover, Choudhury *et al.* (2022) ^90^ reported that the PRS model for sub-Saharan African populations provided higher predictivity power for the LDL-C trait compared to multi-ancestry and European populations.

It is worth reporting that there are several PRS studies that have been done using African populations. However, they are not restricted to sub-Saharan Africa’s populations because the 1,000 genomes reference panel data include samples from Africa populations.

In 2020, Hayat and her colleagues investigated the genetic associations between serum low LDL-cholesterol levels and selected genetics variants in sub-Saharan African of four countries; Kenya, South Africa, Ghana and Burkina Faso. ^93^ Using 1,000 genomes data from the African populations, they selected four genes for their investigation ( *LDLR*, *APOB*, *PCSK9*, and *LDLRAP1*). They performed genotyping of 19 SNPs using 1,000 participants in the Human Heredity and Health in Africa (H3Africa) AWI-Gen Collaborative Center (Africa, Wits-IN-DEPTH Partnership for GENomic studies). Although they used a limited number of variants, the outcome showed a significant association of these SNPs with lower LDL-cholesterol levels in sub-Saharan Africans.

In 2020, Cavazos and Witte proposed the inclusion of variants discovered from various populations to improve PRS transferability to diverse populations. ^94^ They used both simulated data for the Yoruba group of the sub-Saharan African and European populations. They tested their findings on real data consisting of diabetes-free training samples of European ancestry ( *n* = 123,665) and African descent ( *n* = 7,564). They evaluated the performance of PRS analysis using genotype and phenotype data for a test (predictive) data set of European ancestry ( *n* = 394,472) individuals of African origin from the UK Biobank ( *n* = 5,886). Based on their findings, they concluded that incorporating variants selected from the European population will limit the accuracy of PRS values in non-Europeans populations including African communities. Also, they commented on the need for diverse GWAS data to improve PRS accuracy across populations.

In 2017, Márquez-Luna *et al.* ^55^ proposed a multi-ethnic PRS analysis to improve risk prediction in diverse populations including African communities. To overcome the lack of enough training data for the African populations, the authors combined the training data from European samples and training data from the target population. We did not include their study because they did not state whether they used sub-Saharan African communities. This further highlights the challenge of performing PRS analysis in sub-Saharan African populations as a result of insufficient training data.

In 2017, Vassos *et al.* examined PRS values in a group of individuals with first-episode psychosis. ^95^ For the control data set, they combined African-European ( *n* = 70) and a sample of sub-Saharan African ancestries ( *n* = 828). Their finding showed that PRS value was more potent in Europeans, i.e. 9.4% discriminative ability, than in Africans, i.e. only 1.1% discriminative ability in Africans.

PRS analysis is applied to investigate the risk score for prostate cancer. Prostate cancer is considered a complex genetic disease with high heritability which disproportionately affects men of African descent. ^96^ A 1,000 Genomes Project research that included seven African study sites and European males projected the risks of prostate cancer in urban African men. It was determined that the risks of prostate cancer are much more significant in African genomes than European genomes ( *p*-value < 2.2 × 10e-16, Wilcoxon rank-sum test). This continental level pattern is consistent with public health data. ^97^ A further investigation was done by the team of MADCaP (Men of African Descent and Carcinoma of the Prostate Consortium) to study sites that portrayed a substantial amount of overlap in the PRS distributions of different African populations. Based on their findings, the investigators of MADCaP observed within-continent heterogeneity for the predicted risk of prostate cancer. Their findings showed that individuals from Dakar, Senegal have the lowest predicted risks of prostate cancer than other African study sites while individuals from Abuja, Nigeria have the highest predicted risks. The MADCaP team concluded that allele frequency differences at common disease-associated loci can contribute to population-level differences in prostate cancer risk.

## Transferability of PRS on sub-Saharan African populations

Previous studies suggested that PRS derived from individuals of African ancestry performed significantly better in sub-Saharan Africans than PRS derived from individuals of African-Americans and Europeans and multi-ancestry. ^69^^,^^94^^,^^99^^,^^100^ However, PRS might differ across sub-Saharan Africans populations due to differences in contributory role of environmental and genetic factors. For instance, Kamiza *et al.* reported that the differences in environmental and genetic factors play critical roles in transferability of PRS between the South African Zulu and individuals from Ugandan cohort ( Table 5). ^89^ Finding from Kamiza *et al.* noted that the poor performance of PRS across populations has implementation impact in preventative healthcare. Therefore, applying PRS to different ethnic groups even within sub-Saharan Africa may lead to inaccurate result. This further suggests the need for more efforts to optimize polygenic prediction in Africa. For instance, Choudhury *et al.* ^90^ demonstrated that PRS transferability among African can be improved by sample size of the African cohort studies.

**Table 5.** Shows the variability in transferability of PRS on sub-Saharan African populations and the contributory role of environmental factors.

Population	Genetics factors	Environmental factors	Effect of PRS	Transferability
South Africa Zulu, University of KwaZulu Natal	High genetic diversity, which may affect the performance and transferability of PRS within Africa	Urban and rural environmental differences might also be playing a part in the poor transferability of the African American-derived PRS between the Ugandan and South & African Zulu cohorts.	PRS predicted better in the South African Zulu cohort	minor allele frequencies to the poor transferability of the PRS
Ugandan Uganda Genome Resource (UGR), and the phenotypic resource generated from the Uganda General Population Cohort (GPC)	Differences in age, body mass index and allele frequencies. These differences in the performance of PRS in the Ugandan cohort	Urban and rural environmental differences might also be playing a part in the poor transferability of the African American-derived PRS between the Ugandan and South African Zulu cohorts.	Lower in Ugandan cohort	Minor allele frequencies to the poor transferability of the PRS

## Challenges of PRS analysis for the African populations

Many PRS methods have been developed and applied to test the risk score of individuals. Nevertheless, PRS analysis has not been used in the clinical field for the African population. There are still many limitations and challenges regarding the application of PRS analysis in the African population. One of these challenges is lack of sufficient data to perform PRS analysis. For instance, querying the term “sub-Saharan” in the GWAS Catalog repository, the search resulted in only 70 publications out of 4,628 papers. Considering that several publications might use the same GWAS data, we affirm that more GWAS experiments need to be done in sub-Saharan African populations. Lack of African population genetic data might be due to the following reasons: (i) African populations are not well presented in the reference genomes for variant calling and genotype calling; (ii) There is insufficient genetic diversity to capture the African specific variations in the average observable African population, i.e. sample sizes and the number of sub-population representations; (iii) there is lack of infrastructure and funding to perform GWAS experiments in many countries in Africa. Infectious diseases like malaria, tuberculosis, and HIV might still be prioritized by African scientists due to their public health importance and funding opportunities. Providing funding priority for infectious diseases is necessary for African communities as they account for a higher mortality rate in the continent.

Due to a lack of training and test data sets, some scientists choose to use training data from European samples that result in decreased PRS prediction accuracy. Therefore, PRS analysis is not widely applied for clinical utilities in Africa. The theory of genetics stated that when the genetic divergence in the target population and the original GWAS sample increases, the precision of the genetic risk prediction would decline. Several statistical discoveries are linked to this pattern: (i) The discovery of dominant genetic variations in the study population is favored by GWAS; (ii) even when the causative variants are the same, LD yields varied estimates of the marginal effect size for polygenic traits across populations; (iii) population-specific environmental and demographic differences. As a result, given the variety of the African population, the model developed elsewhere for PRS analysis does not fit for African sub-populations. Recent efforts to increase PRS accuracy in non-Europeans have prioritized the European discovered variants and population-specific weighting. Due to a limitation of GWAS studies in African populations, this technique might be utilized to construct an African-specific PRS method that incorporates diverse sources of information. While the African-specific PRS approach aims to improve PRS accuracy, the shortage of long-term funds for GWAS research is another major obstacle in conducting and applying PRS research in the African context. Understudied populations, particularly in Africa provide possibility for genetic research. The common variants in these populations but uncommon or lacking in the European population could not be discovered using European sample sizes. SLC116A11 and HNF1A genes, for example are linked to type 2 diabetes, whereas APOL1 is linked to prostate cancer and end-stage kidney disease in African-Americans. These issues are intractable with statistical techniques alone. Therefore, significant investment is required in African populations to yield similar-sized GWAS of biological traits.

As more data about genetic variation becomes available, the task of increasing the representation of African populations in the GWAS database has become increasingly essential. ^99^^,^^101^ The inclusion of African multi-ethnic groups in GWAS analysis research is crucial for a more thorough, careful genetic variation and interpretation of the underpinnings of complex PRS analysis. ^99^^,^^101^ In comparison to other under-represented populations, the average sample size of GWAS among Europeans continues to expand. PRS analysis in European populations has repeatedly failed to perform in African populations due to LD, confounding of environmental factors across populations and differences in allelic architecture. ^95^^,^^99^^,^^101^^–^^103^ The frequency of causative, risk allele, correlated variants, and disease prevalence all show substantial-frequency variation between populations. ^13^^,^^101^ The magnitude and frequency of disease-causing genetic variants differ greatly among different populations including African ancestry. ^104^ Overcoming these obstacles might lead to an effective clinical management, and specialized therapy for individuals and populations impacted by these complex disease and risk factors all of which would improve the health of those affected. ^99^^,^^104^^,^^105^ Moreover, it could help in decreasing genotype imputation error, increase levels of tag-SNP portability, GWAS design, and effectively addressed GWAS analysis and interpretation in Africa populations. ^101^^,^^104^

Therefore, African state authorities should be made aware of the challenges to make more funds available for genomic research. The funds should not be limited to the research institutes and principal investigators alone but they should equally provide scholarships (postgraduate programs like PhD) and financial aids for young African researchers. We have some promising African research consortiums like The Pan-African Bioinformatics Network for the Human Heredity and Health in Africa (H3ABioNet, *h3abionet.org* ) and the Human Heredity and Health in Africa (H3Africa, *h3africa.org* ) that are contributing in this regard. However, their funds come from outside Africa. There are new regional Africa efforts like the World Bank-funded Africa Center of Excellence (ACE). It is important to state that these initiatives consist of few genomic research projects. A follow-up project to the H3Africa, dedicated to data science health research, entitled Harnessing Data Science for Health Discovery and Innovation in Africa (DS-I Africa) will soon commence.

Moreover, the lack of a pan-African genomic advisory board remains another challenge for genomic research in Africa. The existence of a research advisory board will help with transparency and establish ethical guidelines. These could open the window to get more grants from funding agencies such as the National Center for Biotechnology Information (NCBI). It is clear that without a rigorous ethical guide and transparency policies, it is hard to get long-term funds.

One more challenge of performing PRS for African populations is human migration. Environmental and social factors are the most critical drivers of disease risk than genetics in many cases so they must be effectively addressed. Benton *et al.* ^106^ highlighted that early human migration out of Africa resulted in a higher genetic mutation rate, including disease-associated variants. Therefore, African populations do not carry the variants associated with disease at a higher frequency compared to non-African ancestries. As a result, given the genetic variation resulting from the diverse demographic history of the human populations, PRS prediction accuracy is still insufficient to generalize adequately across different populations, particularly for Africans. ^99^^,^^107^ Furthermore, a lack of diversity in PRS development may contribute to existing health disparities among Africans. ^108^^,^^109^ Therefore, consideration of environmental exposures and evolutionary histories must be key factors when performing PRS analysis.

## Application of PRS analysis on type 2 diabetes in African populations

Diabetes mellitus prevalence was projected in 2019 to be 463 million globally, 4% of which are in African populations. ^110^ In addition, Africa will witness the world’s highest increase in diabetes prevalence by 2045. ^110^^,^^111^ Currently, Africa has the most significant percentage of undiagnosed diabetics (59.7%) in the world. As a result, immediate policies and resources for developing surveillance and an early detection approach to help Africa combat this pandemic has been initiated. ^112^ The use of PRS for the early detection of people who are genetically predisposed to type 2 diabetes could significantly reduce the diabetes burden. According to data from European nations, individuals in the top 90% of the population had a 5.21-fold higher likelihood of developing diabetes than those in the lowest 10%. ^113^ Evidence has shown (coupled with a low GWAS study) that the transferability of polygenic scores developed in Europe decreases accuracy across diverse populations. ^99^ Multi-ethnic PRS could be an alternative. However, the predictive performance of the African Americans and that of multi-ethnic PRS (who has about 80% African admixture) in continental Africans are yet to be examined. ^55^^,^^114^ To ascertain this, Chikowore *et al.* aimed to see how well multi-ethnic, African-Americans, and European PRS would predict type 2 diabetes in Africans. ^112^ For PRS development, the PRSice-2 software was used and the PRS with best result was chosen using area under the curve, i.e AUC and Nagelkerke R2. Finally, the results demonstrated that PRS derived from African Americans outperformed both multi-ethnic and European PRS in predicting type 2 diabetes. An earlier study of type 2 diabetes based on genetic risk score in Black South Africans used weight from Europeans (OR = 1.21, 95%CI). ^2^ However, due to weights obtained from European-only studies, limited sample size, and use of only genotyped SNPs, this research was less predictive of Type 2 diabetes. Unlike previous work, this current study (Fatumo *et al.* ^112^) took advantage of a larger sample size (1,690), improved genome coverage and a multi-ethnic discovery dataset GWAS. All of these factors worked together to improve the PRS predictive ability. ^2^

## PRS analysis on breast and prostate cancers in the continent of Africa

Africa reportedly has the highest age-standardized death rate of breast cancer globally with sub-Saharan Africa having the highest prevalence rates. Although the occurrence in Africa was lower than in other continents, except for Asia, the mortality rate in Africa’s sub-Saharan region (for example in Nigeria) was the highest in the world. ^115^ Men of African origin have a greater prevalence and mortality rate from prostate cancer than men of other ethnic groups. Uganda has one of the highest prostate cancer incidence rates of all African nations. ^116^ Genetic contributions to this difference are supported by evidence of genetic heterogeneity across populations. Breast and prostate cancer research in African populations can contribute to the elevated disease burden within this population by genetic risk factors. As a result, policymakers, academics and the general public must become aware of the rising threat that breast and prostate cancer can pose to Africa’s growth. Early detection and stratification of women and men based on their risk of breast and prostate cancer using PRS could enhance screening and prevention strategies. Early detection of high disease risk individuals could also reduce the burden and threat to Africa’s development. The application of PRS for breast and prostate cancer allows for early detection and risk stratification for recommendations and monitoring. ^117^ To date, most of the GWAS SNPs were found almost entirely in European ancestry populations. They also demonstrate distinct patterns of relationship among the African populace. ^17^^,^^117^ In addition, variants found in one community often do not apply to other populations of African ancestry. ^118^ These contradictory findings may be attributed to various factors, including variations in allele frequencies and LD and differences in population characteristics within one ethnicity. As a result, there is a risk of PRS transferring PRS across populations. ^119^ Some studies investigate PRS developed using GWAS data from various ancestry groups. ^120^^,^^121^ For example, Belsky *et al.* ^120^ constructed an obesity-based PRS relying on GWAS from European ancestry and discovered that it performed poorly in African Americans but worked well in European ancestry. ^120^ On the other hand, Fritsche *et al.* ^118^ concluded that, to some degree, cancer based PRS obtained from large Europeans ancestry GWAS may still be employed for disease risk stratification in populations if the limitations listed below are properly addressed:

• To accurately put an individual’s PRS within their reference PRS distributions, a matched ancestry cohort with large control sample sizes is required.
• Non-European ancestry-derived PRS will be particularly useful for breast and prostate cancers because they have certain advantages over other traits: the high heritability is relatively high, normal in all ancestry groups, and publicity of summary statistics.
• Unlike individuals of diverse ancestries from different populations, the participants in the UK Biobank are mostly from the same country and healthcare accessibility and other risk factors are similar.

If summary statistics and large GWAS are available, Fritsche *et al.* ^116^ argued that PRS development based on the same ancestral group might increase its predictive ability if summary statistics and large GWAS are available. Several methods are now being investigated to increase PRS predictive accuracy in African populations. If a large-scale GWAS for non-European populations are unavailable, these methods might be employed to improve PRS. On the other hand, these methods may incorporate the fact that SNP selection based on European based GWAS is applicable when employing European based GWAS effect sizes in ethnically mismatched populations. ^74^^,^^116^

## Conclusion and future research

There are several approaches under the umbrella of PRS analysis. GWAS are conducted on finite samples extracted from particular subsets of the human population. Moreover, the SNP effect size estimates are some combination of true effect and stochastic variation, thus producing’winner’s curse’ among the top-ranking associations and the estimated effects may not be well generalized to different populations. Furthermore, the correlation complicates the aggregation of SNP effects across the genome. Therefore, linkage disequilibrium holds the key to apply PRS analysis across ethnic groups. Thus, critical factors in the development of methods for calculating PRS values are

• The potential adjustment of GWAS estimated effect sizes e.g. via shrinkage and incorporation of their uncertainty.
• The tailoring of PRS values to target populations.
• The task of dealing with LD.

As members of the H3Africa consortium and the Associated Bioinformatics Consortium, H3ABioNet, (see h3abionet.org and https://sysbiolpgwas.waslitbre.org), we are working to extend existing methods to be applicable to African populations. Also, one future direction will be to develop an African-specific PRS method that combines the different sources of information. The information that we would consider to improve the current PRS methods include: (i) individual’s ancestry information to include the diversity within sub-Saharan populations; (ii) environmental risk factors to include the environmental diversity in Africa. Due to the variation in genetic architecture among trans-ethnic groups, we will consider incorporating information at the transcriptome level in the sub-Saharan populations. Thus, providing a new PRS method that performs individual ancestry estimation and transcriptome risk score would improve the predictive value of the PRS besides providing insights into the molecular determinants of phenotypic traits, including rare diseases.

## Data availability

### Underlying data

No data is associated with this article.

### Extended data

Dryad: Polygenic Risk Score in Africa Populations: Progress and challenges, https://doi.org/10.5061/dryad.hdr7sqvk8. ^122^

This project contains the following extended data:

• README file which provides information about the contents of the other file.
• A table contains selected studies in 2020 that demonstrate the PRS methods applied to diabetes type II, prostate cancer, and breast cancer.

Data are available under the terms of the Creative Commons Zero “No rights reserved” data waiver (CC0 1.0 Public domain dedication).
