# Supplementary material for: Polygenic Risk Score in African populations: progress and challenges
Source: F1000Res. 2023 Apr 11;11:175. Originally published 2022 Feb 14. [Version 2] doi: 10.12688/f1000research.76218.2 (PMC10233318; doi:10.12688/f1000research.76218.2)
Supplement: Peer review history

# Peer review: Reviewer response for version 2

**DOI:** 10.5256/f1000research.143350.r169138

**Published:** 2023-05-31

**Authors:** Bingxin Zhao

**Affiliations:** 1 Department of Statistics and Data Science, University of Pennsylvania, Philadelphia, Pennsylvania, USA

I would like to thank the authors for thoroughly addressing my comments.

Is the review written in accessible language?

Yes

Are all factual statements correct and adequately supported by citations?

Yes

Are the conclusions drawn appropriate in the context of the current research literature?

Yes

Is the topic of the review discussed comprehensively in the context of the current literature?

Yes

Reviewer Expertise:

genetics and statistics

I confirm that I have read this submission and believe that I have an appropriate level of expertise to confirm that it is of an acceptable scientific standard.

# Peer review: Reviewer response for version 2

**DOI:** 10.5256/f1000research.143350.r169137

**Published:** 2023-04-24

**Authors:** Cathryn M. Lewis, Michelle Kamp

**Affiliations:** 1 Social, Genetic and Developmental Psychiatry Centre & Department of Medical and Molecular Genetics, King's College London, London, UK; 2 Sydney Brenner Institute for Molecular Bioscience, University of the Witwatersrand Johannesburg, Johannesburg, Gauteng, South Africa

The authors have fully responded to our comments - thank you. We enjoyed reading the paper and it will make a good addition to the literature in this field.

Is the review written in accessible language?

Yes

Are all factual statements correct and adequately supported by citations?

Yes

Are the conclusions drawn appropriate in the context of the current research literature?

Yes

Is the topic of the review discussed comprehensively in the context of the current literature?

Yes

Reviewer Expertise:

Polygenic scores

We confirm that we have read this submission and believe that we have an appropriate level of expertise to confirm that it is of an acceptable scientific standard.

# Peer review: Reviewer response for version 1

**DOI:** 10.5256/f1000research.80186.r137670

**Published:** 2022-06-22

**Authors:** Cathryn M. Lewis

**Affiliations:** 1 Social, Genetic and Developmental Psychiatry Centre & Department of Medical and Molecular Genetics, King's College London, London, UK

**General:**

 Adam *et al. *provide an extensive review of the important topic of PRS methods, and their applications in African ancestry populations. The paper summarises the various approaches to calculating PRS and provides a fair assessment of the advantages and disadvantages of each method. It also describes the challenges associated with calculating PRS in African populations and the approaches currently being undertaken to address these. The paper is comprehensive and a useful addition to the literature, pulling together a large amount of information across methods for calculating polygenic scores, and their applications in African populations.

**Major comments:**

 To make the application of polygenic scores more accessible the authors could summarise the findings from studies conducted in Sub-Saharan African populations. For example, a table that orders studies by outcome/disease type and summarises key study parameters: methods (cohort/populations, LD reference panel, method) and results (variance explained) would be useful to readers.

 When referring to predictive power being limited in African populations, additional detail as to what the AUC or equivalents are would be useful to contextualize the scores and provide comparisons to scores in non-African populations e.g. scores in EUR and AFR for a similar trait.

 We suggest that the authors could also address the variability in transferability of scores not only between super-populations (eg. AFR and EUR) but also between SSA populations and the potential contributory role of environmental factors. Potential paper to do this includes Kamiza, A.B. *et al.* Transferability of genetic risk scores in African populations. ^1^

 One of the most recent useful advances in PRS development for ancestrally diverse populations is the PRS-CSx method. ^2^ Although this new method was published after the date cut-off for the manuscript, a comment could be added in the Discussion. 

**Minor comments:**

 Introduction:

- Table 1 is a comprehensive summary of PRS tools. How are these ordered? Given the authors have previously classified the methods into four groups (p3), it might be useful to use this information in structuring the table. In addition, what parameters/factors were used to determine whether an approach was user-friendly?
- P3 “PRS analysis is used to predict an individual heritability by incorporating all selected SNPs.” What do the authors mean here by the phrase ‘individual heritability’? PRS methods that incorporate LD. In practice, when the markers are LD pruned…

PRS analysis on African populations:

- This number represents about 4.553483% of total hits that - this number represents about 4.55% or 4.6% of total hits that
- They observed that the predictive power of genetic risk scores was higher among African Americans (n=9139) and European Americans (n=9594) relative to the sub-Saharan African populations (n=5200). 
  - Consistency in number formatting: They observed that the predictive power of genetic risk scores was higher among African Americans (n=9139) and European Americans (n=9594) relative to the sub-Saharan African populations (n=5200).

Is the review written in accessible language?

Yes

Are all factual statements correct and adequately supported by citations?

Yes

Are the conclusions drawn appropriate in the context of the current research literature?

Yes

Is the topic of the review discussed comprehensively in the context of the current literature?

Yes

Reviewer Expertise:

Statistical Genetics, Genetic epidemiology

I confirm that I have read this submission and believe that I have an appropriate level of expertise to confirm that it is of an acceptable scientific standard, however I have significant reservations, as outlined above.

## References

[1] : Transferability of genetic risk scores in African populations. *Nat Med* .2022;28(6) : 10.1038/s41591-022-01835-x 1163-1166 10.1038/s41591-022-01835-x 35654908PMC9205766

[2] : Improving polygenic prediction in ancestrally diverse populations. *Nat Genet* .2022;54(5) : 10.1038/s41588-022-01054-7 573-580 10.1038/s41588-022-01054-7 35513724PMC9117455

# Response

**Published:** 2023-01-17

**Authors:** Ezekiel Adebiyi

**Affiliations:** Covenant University, Nigeria

**Answers from the authors to the reviewer’s comments**

 Authors would like to thank the reviewer for valuable comments and suggestions. Below are the answers for each reviewer’s comments:

**Major comments responses**

**1. To make the application of polygenic scores more accessible the authors could summarise the findings from studies conducted in Sub-Saharan African populations. For example, a table that orders studies by outcome/disease type and summarises key study parameters: methods (cohort/populations, LD reference panel, method) and results (variance explained) would be useful to readers.**

 Authors thank the reviewer for his suggestions. Authors think that this point is quite similar to a comment raised by reviewer 1. Therefore, we address these two comments together by adding Table (4), **pages 17-19.**

**2. When referring to predictive power being limited in African populations, additional detail as to what the AUC or equivalents are would be useful to contextualize the scores and provide comparisons to scores in non-African populations e.g. scores in EUR and AFR for a similar trait.**

 Authors thank the reviewer for his suggestions, and we agree that adding AUC as a method for evaluating PRS method will be useful for readers who are not familiar with machine learning and model evaluation. Therefore, we addressed this point by adding a text about AUC in the main manuscript.

**3. We suggest that the authors could also address the variability in transferability of scores not only between super-populations (eg. AFR and EUR) but also between SSA populations and the potential contributory role of environmental factors. Potential paper to do this includes Kamiza, A.B. et al. Transferability of genetic risk scores in African populations**

 Authors accepted the reviewer's comment, we added the text below on **page 19** of the revised version. We also added table 5 to give a summary of  Transferability of PRS.  in African populations.

 "Previous studies suggested that PRS derived from individuals of African ancestry performed significantly better in sub-Saharan Africans than PRS derived from individuals of African Americans and Europeans and multiancestry (Duncan et al., 2019; Cavazos & Witte, 20210; Martin et al., 2019; Johnson et al., 2015). However, PRS might differ across sub-Saharan Africans populations due to differences in contributory role of environmental and genetic factors. For instance, Kamiza et al} reported that the differences in environmental and genetic factors play critical roles in transferability of PRS between the South African Zulu and individuals from Ugandan cohort Kamiza et al (2022), Table 5.. Finding from Kamiza et al} noted that the poor performance of PRS across populations has implementation impact in preventative healthcare. Therefore, applying PPRS to different ethnic groups, even within within sub-Saharan Africa, may lead to inaccurate result. This further suggests the need for more efforts to optimize polygenic prediction in Africa. For instance, Choudhury et al (2022) demonstrated that PRS transferability among African can be improved by sample size of the African cohort studies."

**References**

 Duncan, L., Shen, H., Gelaye, B. et al. Analysis of polygenic risk score usage and performance in diverse human populations. Nat Commun 10, 3328 (2019). https://doi.org/10.1038/s41467-019-11112-0

 Cavazos, T. B., & Witte, J. S. (2021). Inclusion of variants discovered from diverse populations improves polygenic risk score transferability. HGG advances, 2(1), 100017. https://doi.org/10.1016/j.xhgg.2020.100017

 Martin, A. R., Kanai, M., Kamatani, Y., Okada, Y., Neale, B. M., & Daly, M. J. (2019). Clinical use of current polygenic risk scores may exacerbate health disparities. Nature genetics, 51(4), 584–591. https://doi.org/10.1038/s41588-019-0379-x

 Johnson, L., Zhu, J., Scott, E. R., & Wineinger, N. E. (2015). An Examination of the Relationship between Lipid Levels and Associated Genetic Markers across Racial/Ethnic Populations in the Multi-Ethnic Study of Atherosclerosis. PloS one, 10(5), e0126361. https://doi.org/10.1371/journal.pone.0126361

 Kamiza AB, Toure SM, Vujkovic M, Machipisa T, Soremekun OS, Kintu C, Corpas M, Pirie F, Young E, Gill D, Sandhu MS, Kaleebu P, Nyirenda M, Motala AA, Chikowore T, Fatumo S. Transferability of genetic risk scores in African populations. Nat Med. 2022 Jun;28(6):1163-1166. doi: 10.1038/s41591-022-01835-x. Epub 2022 Jun 2. PMID: 35654908; PMCID: PMC9205766.

 4 **. One of the most recent useful advances in PRS development for ancestrally diverse populations is the PRS-CSx method.2 Although this new method was published after the date cut-off for the manuscript, a comment could be added in the Discussion.**

 Authors thank the reviewer for the comment and we agree that PRS-CSx method is on the key method that can be used for the application of PRS across multi-ethnic group. We did not include because we submitted our review before publishing PRS-CSx method. However, we have include an overview of the PRS-CSx method in our reviewed manuscript, **page 11**. We also cited the article for those are interested to know more about its underlying algorithm.

**PRS-CSx method**

 PRS-CSx method is proposed to improve the accuracy of the application of PRS across multi-ethnic populations by using posterior inference algorithm (Ruan et al., 2022; Ge et al., 2022). PRS-CSx combines GWAS summary files from different population to increase the accuracy of PRS. PRS-CSx estimates population-specific effect size by incorporating the population-specific LD pattern, population-specific allele frequency information, and the information of shared continuous shrinkage prior across populations. For more details about the mathematical method underlying PRS-CSx, refer to Ruan et al., 2022).

**References**

 Ruan, Y., Lin, YF., Feng, YC.A. *et al.* Improving polygenic prediction in ancestrally diverse populations. *Nat Genet* **54**, 573–580 (2022). https://doi.org/10.1038/s41588-022-01054-7

 Ge, T., Irvin, M. R., Patki, A., Srinivasasainagendra, V., Lin, Y. F., Tiwari, H. K., Armstrong, N. D., Benoit, B., Chen, C. Y., Choi, K. W., Cimino, J. J., Davis, B. H., Dikilitas, O., Etheridge, B., Feng, Y. A., Gainer, V., Huang, H., Jarvik, G. P., Kachulis, C., Kenny, E. E., … Karlson, E. W. (2022). Development and validation of a trans-ancestry polygenic risk score for type 2 diabetes in diverse populations. *Genome medicine*, *14*(1), 70. https://doi.org/10.1186/s13073-022-01074-2

**Minor comments:**

- **Table 1 is a comprehensive summary of PRS tools. How are these ordered? Given the authors have previously classified the methods into four groups (p3), it might be useful to use this information in structuring the table. In addition, what parameters/factors were used to determine whether an approach was user-friendly?**

The authors thank the reviewer for the comments. We determined whether an approach is user-friendly based on the installation process, the popularity of the methods among the users, the availability of an application manual (tutorial), the application user interface, and the number of options that should be considered and tuned by users. However, we have removed a column in the table (no.) that classifies the methods from the revised version based on four different approaches, such as (p3): clumping with thresholding (C+T), p-value thresholding, penalized regression, and Bayesian shrinkage, because some tools perform PRS analysis using more than one method. For instance, LDpred can perform PRS analysis either using clumping and thresholding (C+T) or the p-value thresholding method.

 We also cited et al . (2022), which performed a recent a comparison on some of these tools.

**References**

 Wang Y, Tsuo K, Kanai M, Neale BM, Martin AR. Challenges and Opportunities for Developing More Generalizable Polygenic Risk Scores. Annu Rev Biomed Data Sci. 2022 Aug 10;5:293-320. doi: 10.1146/annurev-biodatasci-111721-074830. Epub 2022 May 16. PMID: 35576555.

  

- **P3 “PRS analysis is used to predict an individual heritability by incorporating all selected SNPs.” What do the authors mean here by the phrase ‘individual heritability’? PRS methods that incorporate LD. In practice, when the markers are LD pruned…**

We mean by individual heritability here by the proportion of trait variance (phenotype) that is associated with genetic variants (genotype). We cited Privé et al (2020) & Vilhjálmsson et al (2015) that provided more details of individual heritability that can be explained by the genetic variants.

**References**

 Florian Privé, Julyan Arbel, Bjarni J Vilhjálmsson, LDpred2: better, faster, stronger, Bioinformatics, Volume 36, Issue 22-23, 1 December 2020, Pages 5424–5431, https://doi.org/10.1093/bioinformatics/btaa1029

 Vilhjálmsson BJ, Yang J, Finucane HK, Gusev A, Lindström S, Ripke S, Genovese G, Loh PR, Bhatia G, Do R, Hayeck T, Won HH; Schizophrenia Working Group of the Psychiatric Genomics Consortium, Discovery, Biology, and Risk of Inherited Variants in Breast Cancer (DRIVE) study, Kathiresan S, Pato M, Pato C, Tamimi R, Stahl E, Zaitlen N, Pasaniuc B, Belbin G, Kenny EE, Schierup MH, De Jager P, Patsopoulos NA, McCarroll S, Daly M, Purcell S, Chasman D, Neale B, Goddard M, Visscher PM, Kraft P, Patterson N, Price AL. Modeling Linkage Disequilibrium Increases Accuracy of Polygenic Risk Scores. Am J Hum Genet. 2015 Oct 1;97(4):576-92. doi: 10.1016/j.ajhg.2015.09.001. PMID: 26430803; PMCID: PMC4596916.

- **PRS analysis on African populations: This number represents about 4.553483% of total hits that - this number represents about 4.55% or 4.6% of total hits that**

Authors thank the reviewer for this comment. We updated the Pubmed search terms that we used and formatted all numbers accordingly.

- **They observed that the predictive power of genetic risk scores was higher among African Americans (n=9139) and European Americans (n=9594) relative to the sub-Saharan African populations (n=5200). - Consistency in number formatting: They observed that the predictive power of genetic risk scores was higher among African Americans (n=9139) and European Americans (n=9594) relative to the sub-Saharan African populations (n=5200).**

Authors thank the reviewer for this comment. We formatted the style of all numbers accordingly.

# Peer review: Reviewer response for version 1

**DOI:** 10.5256/f1000research.80186.r124444

**Published:** 2022-03-10

**Authors:** Bingxin Zhao

**Affiliations:** 1 Department of Statistics and Data Science, University of Pennsylvania, Philadelphia, Pennsylvania, USA

This is an interesting paper on the review of PRS, with a focus on African populations. The research question is interesting and the writing is knowledgeable. I have the following suggestions that might improve the quality of this paper. 

**Major:**

1. Although the title states that this paper focuses on African populations, I feel the current version of this paper is a bit too focused on general PRS research in all populations. I encourage the authors to include more details and examples of PRS applications in African populations, especially sub-Saharan African communities. Then, the paper may be more balanced. 
  For example, in Section "PRS analysis on African populations": *The traits studied using PRS analysis in African populations include types 1 & 2 diabetes mellitus, depression, ischemic stroke, schizophrenia, sarcoidosis, Alzheimer’s disease, obesity, insomnia disorder, post-traumatic stress and cancer. Undermentioned are some selected PRS studies in sub-Saharan African populations.*
  The citations of these studies could be provided in the paper or a supplemental table. Then, the authors could provide a general overview of all the findings of these papers in the main text or supplemental note.
  This is just one example, the authors could consider improve other sections of the paper to extend the discussions of African populations. 
2. In the "The predictive power of PRS analysis" section, the authors could discuss and include a few more recent studies on the mathematical properties of PRS, such as https://onlinelibrary.wiley.com/doi/10.1111/biom.13466 and https://arxiv.org/abs/1911.10142. Particularly, https://arxiv.org/abs/1911.10142 studies the cross-population accuracy, which may fit the topic and discussion paper well. 

**Minor:**

1. In the beginning of the "PRS methods that incorporate LD" section, the "W" in "When" should be lower case?
2. In the sentence " *Feng & Smoller 24presented the PRS-CS-auto method, a fully Bayesian approach that enables automatic learning of..*." The citation (24) of Feng and Smoller is wrong?
3. " *For instance, searching PubMed for PRS in African populations on August 21, 2021 (see Figure 1 and Box 1), only gave 8,843 hits.*" What is the result for sub-Saharan African?
4. " *This number represents about 4.553483% of total hits that*" 4.55% could be enough here. 

Is the review written in accessible language?

Yes

Are all factual statements correct and adequately supported by citations?

Yes

Are the conclusions drawn appropriate in the context of the current research literature?

Yes

Is the topic of the review discussed comprehensively in the context of the current literature?

Yes

Reviewer Expertise:

genetics and statistics

I confirm that I have read this submission and believe that I have an appropriate level of expertise to confirm that it is of an acceptable scientific standard, however I have significant reservations, as outlined above.

## References

[1] : On polygenic risk scores for complex traits prediction. *Biometrics* .2021; 10.1111/biom.13466 10.1111/biom.13466 33786811

# Response

**Published:** 2023-01-17

**Authors:** Ezekiel Adebiyi

**Affiliations:** Covenant University, Nigeria

**Answers from the authors to the reviewer’s comments**

 Authors would like to thank the reviewer for valuable comments and suggestions. Below are the answers for each reviewer’s comments:

**Our Response to the Major Comments**

**1. Although the title states that this paper focuses on African populations, I feel the current version of this paper is a bit too focused on general PRS research in all populations. I encourage the authors to include more details and examples of PRS applications in African populations, especially sub-Saharan African communities. Then, the paper may be more balanced.**

**For example, in Section "PRS analysis on African populations": The traits studied using PRS analysis in African populations include types 1 & 2 diabetes mellitus, depression, ischemic stroke, schizophrenia, sarcoidosis, Alzheimer’s disease, obesity, insomnia disorder, post-traumatic stress and cancer. Undermentioned are some selected PRS studies in sub-Saharan African populations.**

**The citations of these studies could be provided in the paper or a supplemental table. Then, the authors could provide a general overview of all the findings of these papers in the main text or supplemental note.**

**This is just one example, the authors could consider improve other sections of the paper to extend the discussions of African populations.**

 The Authors thank the reviewer for this major comment and we agree totally with the reviewer’s point of view. Therefore, we have reviewed the content and also changed the title-heading **“PRS Analysis on African Populations” to** more specific title-header **“PRS Analysis on Sub-Saharan African Populations”, page 15**. More so, we have cited the key articles and summarized the findings in the main text of the manuscript while we included additional table content, **pages 17-19**. The following text is provided for the revised version of  the manuscript.

**PRS Analysis on Sub-Saharan African Populations**

 The PRS Analysis on Sub-Saharan African populations is limited due to lack of enough GWAS studies on traits associated with Sub-Saharan African populations. For instance, searches on PubMed for PRS on Sub-Saharan African populations on December 23, 2022 (see Figure 1 and Box 1) results in only 5 hits (4 research articles and 1 review paper). These four research articles performed PRS analysis mainly on traits associated with cardiometabolic disease such as heart attack, type 2 diabetes, and stroke. Other contributing risk factors including body mass index (BMI), waist circumference (WC), hip circumference (HC), waist-to-hip ratio (WHR), systolic blood pressure (SBP), diastolic blood pressure (DBP), triglycerides (TG), total cholesterol (TC), low-density lipoprotein(LDL), high-density lipoprotein (HDL), fasting plasma glucose( FPG), and type 2 diabetes (T2D), low-density lipoprotein cholesterol (LDL-C), high-density lipoprotein cholesterol (HDL-C), triglycerides (TGs) and total cholesterol (TC) (Ekoru et al., 2021; Kamiza et al., 2022; Choudhury et al., 2022; Meeks et al., 2022). More so, the variance detected for Sub-Saharan populations in these studies has been summarized in Table 4.

 The general outcome of these five articles emphasize an urgent needs of GWAS research studies for Sub-Saharan African populations in order to continue to perform PRS analysis that would add more benefit to the use of PRS in precision medicine as well as an improved representation of multiple ethnic populations in GWAS to better reflect risk stratification, variabilities in genetic equitable, and translation of GRS in clinical setting . For instance, Ekoru et al (2021) demonstrated that several traits such as cardiometabolic have less predictive power of genetics risk score in Sub-Saharan Africans compared to others populations such as African Americans and European Americans. The less predictive power of cardiometabolic traits were as a result of underrepresented African populations based on GWAS data in the current reference genomes. However, Kamiza et al. (2022) studies showed an increase in PRS performance on lipid traits (such as, LDL-C) with dataset from Sub-Saharan populations, European, and multi-ancestry. Other lipid traits include HDL-C, TGs and TC. PRSs performance varies significantly even among the sub-Saharan African populations. This variation on PRS performance occurs due to variations on Africa population-specific genetic structure, such as minor allele frequencies and the population-specific associated environmental factors.

 It is worth reporting that there are several PRS studies that have been done using African populations. However, these studies are not restricted to sub-Saharan Africa's populations because the 1000 genomes reference panel data include samples from Africa populations. In 2020, Hayat and her colleagues investigated the genetic associations between serum low LDL-cholesterol levels and selected genetic variants (Hayat et al., 2020). Using 1000 genomes data from the African populations, they selected four genes for their investigation (LDLR, APOB, PCSK, and LDLRAP1). They performed genotyping of 19 SNPs using 1000 participants in the Human Heredity and Health in Africa (H3Africa) AWI-Gen Collaborative Center (Africa, Wits-IN-DEPTH Partnership for GENomic studies). Although they used a limited number of variants, the outcome showed a significant association of these SNPs with lower LDL-C levels in sub-Saharan Africans.

 In 2020, Cavazos and Witte proposed the inclusion of variants discovered from various populations to improve PRS transferability to diverse populations (Cavazos and Witte, 2020). They used both simulated data for the Yoruba group of the sub-Saharan African and European populations. They tested their findings on real data consisting of diabetes-free training samples of European ancestry (n = 123,665) and African descent (n = 7564).They evaluated the performance of PRS analysis using genotype and phenotype data for a test (predictive) data set of European ancestry (n = 394472) individuals of African origin from the UK Biobank (n = 5886). Based on their findings, they concluded that incorporating variants selected from the European population will limit the accuracy of PRS values in non-Europeans populations including African communities. Also, they commented on the need for diverse GWAS data to improve PRS accuracy across populations.

 In 2017, Marquez-Luna et al. (2017) proposed a multi-ethnic PRS analysis to improve risk prediction in diverse populations including African communities. To overcome the lack of enough training data for the African populations, the authors combined the training data from European samples and training data from the target population. We did not include their study because they did not state whether they used sub-Saharan African communities. This further highlights the challenge of performing PRS analysis in sub-Saharan African populations as a result of insufficient training data.

 In 2017, Vassos et al. (2017) examined PRS values in a group of individuals with first-episode psychosis (Vassos et al., 2017). For the control data set, they combined African-European (n= 70) and a sample of sub-Saharan African ancestries (n=828). Their finding showed that PRS value was more potent in Europeans, i.e. 9.4% discriminative ability, than in Africans, i.e. only 1.1% discriminative ability in Africans.

**References**

 Badianyama, M., Mpanya, D., Adamu, U., Sigauke, F., Nel, S. and Tsabedze, N., 2022. New biomarkers and their potential role in heart failure treatment optimisation—An African perspective. Journal of Cardiovascular Development and Disease, 9(10), p.335.

 Ekoru K, Adeyemo AA, Chen G, Doumatey AP, Zhou J, Bentley AR, Shriner D, Rotimi CN. Genetic risk scores for cardiometabolic traits in sub-Saharan African populations. Int J Epidemiol. 2021 Aug 30;50(4):1283-1296. doi: 10.1093/ije/dyab046. PMID: 33729508; PMCID: PMC8407873.

 Kamiza AB, Toure SM, Vujkovic M, Machipisa T, Soremekun OS, Kintu C, Corpas M, Pirie F, Young E, Gill D, Sandhu MS, Kaleebu P, Nyirenda M, Motala AA, Chikowore T, Fatumo S. Transferability of genetic risk scores in African populations. Nat Med. 2022 Jun;28(6):1163-1166. doi: 10.1038/s41591-022-01835-x. Epub 2022 Jun 2. PMID: 35654908; PMCID: PMC9205766.

 Choudhury A, Brandenburg JT, Chikowore T, Sengupta D, Boua PR, Crowther NJ, Agongo G, Asiki G, Gómez-Olivé FX, Kisiangani I, Maimela E, Masemola-Maphutha M, Micklesfield LK, Nonterah EA, Norris SA, Sorgho H, Tinto H, Tollman S, Graham SE, Willer CJ; AWI-Gen study; H3Africa Consortium, Hazelhurst S, Ramsay M. Meta-analysis of sub-Saharan African studies provides insights into genetic architecture of lipid traits. Nat Commun. 2022 May 11;13(1):2578. doi: 10.1038/s41467-022-30098-w. Erratum in: Nat Commun. 2022 Aug 2;13(1):4474. PMID: 35546142; PMCID: PMC9095599.

 Meeks KAC, Bentley AR, Doumatey AP, Adeyemo AA, Rotimi CN. Mendelian randomization study reveals a causal relationship between adiponectin and LDL cholesterol in Africans. Sci Rep. 2022 Nov 8;12(1):18955. doi: 10.1038/s41598-022-21922-w. PMID: 36347891; PMCID: PMC9643497.

**2.  In the "The predictive power of PRS analysis" section, the authors could discuss and include a few more recent studies on the mathematical properties of PRS, such as https://onlinelibrary.wiley.com/doi/10.1111/biom.13466 and https://arxiv.org/abs/1911.10142. Particularly, https://arxiv.org/abs/1911.10142 studies the cross-population accuracy, which may fit the topic and discussion paper well.**

 Authors thank the reviewer for his valuable suggestion. We added the text below to the manuscript, **page 13**, to improve it.

 “Zhao & Zou (2022) showed in their study that PRS predictivity can be improved based on SNPs selection. The process of SNPs selection depends on the genetic architecture, i.e, causal variants, and the sample size of the training data set. To select a set of SNPs that provide the optimal PRS prediction, the sample size of the training data set should be much larger than the number of potential causal variants. That is, performing PRS where the ratio of causal variants and sample size is large results in poor PRS prediction due failure in causal variants separations. Therfores, in the case of the ratio of causal variants to the sample size is large, i.e, small sample size is the training data set, Zhao & Zou recommended to include a large number of variants to get higher PRS prediction power. Moreover, Zhao & Zou recommended to include independent uncorrelated variants to improve PRS predictivity. Moverover, Zhao et al. (2022) demonstrated that accounting for correlation between causal variants, i.e, LD, will improve PRS predictivity and accuracy for heterogeneous populations.

 Furthermore, the performance of the PRS mathematical model can be assessed by evaluating the model's output using machine learning techniques, including area under the curve (AUC) of the receiver operating characteristic (ROC) (Janssens et al., 2007; Igo et al., 2019). The ROC can be visualized by plotting true positive rate against false positive rate for model’s thresholds. Janssens et al. (2007) recommend using a model that provides AUC >0.75 for PRS clinical utility, ie, screening of individuals who are at risk. Igo et al. (2019) has suggested using the proportion of trait variability explained by one or more variants as an indicator for PRS predictivity, for more details refer to Janssens et al. (2007).

 Igo, R. P., Jr, Kinzy, T. G., & Cooke Bailey, J. N. (2019). Genetic Risk Scores. *Current protocols in human genetics*, *104*(1), e95. https://doi.org/10.1002/cphg.95

 Janssens, A. C., Moonesinghe, R., Yang, Q., Steyerberg, E. W., van Duijn, C. M., & Khoury, M. J. (2007). The impact of genotype frequencies on the clinical validity of genomic profiling for predicting common chronic diseases. Genetics in Medicine, 9, 528–535. doi: 10.1097/GIM.0b013e31812eece0.

 Zhao, B., & Zou, F. (2022). On polygenic risk scores for complex traits prediction. *Biometrics*, *78*(2), 499–511. https://doi.org/10.1111/biom.13466

 Zhao, B., Zou, F., & Zhu, H. (2022). Cross-trait prediction accuracy of summary statistics in genome-wide association studies. *Biometrics*, 10.1111/biom.13661. Advance online publication. https://doi.org/10.1111/biom.13661

**Authors responses to the Minor comment**

**1. In the beginning of the "PRS methods that incorporate LD" section, the "W" in "When" should be lower case?**

 Authors thank the reviewers for the suggestions, and we applied the correction accordingly.

**2. In the sentence "Feng & Smoller 24presented the PRS-CS-auto method, a fully Bayesian approach that enables automatic learning of..." The citation (24) of Feng and Smoller is wrong?**

 Authors thank the reviewers for the suggestions, and we applied the correction accordingly. We cited it as Ge et a as given is the correct citation below

 Ge T, Chen C-Y, Ni Y, Feng Y-CA, Smoller JW. Polygenic prediction via Bayesian regression and continuous shrinkage priors. Nat Commun. 2019;10(1):1776.

**3. "For instance, searching PubMed for PRS in African populations on August 21, 2021 (see Figure 1 and Box 1), only gave 8,843 hits." What is the result for sub-Saharan African?**

 Authors thank the reviewers for the suggestions, we updated our search terms and we have added the recent results, including PubMed hits for sub-Saharan African in Figure 1.

**4. "This number represents about 4.553483% of total hits that" 4.55% could be enough here.**

 We thank the reviewer, such small number demonstrate the lack of enough PRS studies on African populations.
